# LC-MS Profiling and Biological Activity of Unexplored *Leucas nubica* Benth. (Lamiaceae)

**DOI:** 10.3390/plants15040522

**Published:** 2026-02-07

**Authors:** Dimitrina Zheleva-Dimitrova, Gokhan Zengin, Sakina Yagi, Solafa Suliman, Reneta Gevrenova

**Affiliations:** 1 Department of Pharmacognosy, Faculty of Pharmacy, Medical University, 1000 Sofia, Bulgaria; rgevrenova@pharmfac.mu-sofia.bg; 2 Physiology and Biochemistry Research Laboratory, Department of Biology, Science Faculty, Selcuk University, Konya 42130, Turkey; gokhanzengin@selcuk.edu.tr; 3 Department of Botany, Faculty of Science, University of Khartoum, Khartoum P.O. Box 321, Sudan; sakinayagi@gmail.com (S.Y.); solafa.suliman@gmail.com (S.S.)

**Keywords:** LC-MS profiling, *Leucas nubica* Benth., antioxidant activity, enzyme inhibitory activity

## Abstract

*Leucas nubica* Benth. (Lamiaceae) is an annual herbaceous plant, native to east and northeast tropical Africa. The whole plant is renowned for the treatment of jaundice. The present study aimed at an in-depth phytochemical profiling and evaluation of in vitro antioxidant and enzyme inhibitory potential of methanol–aqueous extract from *L. nubica* aerial parts. The liquid chromatography–high-resolution mass spectrometry (LC-HRMS) experiment revealed more than 70 secondary metabolites, including carboxylic and phenolic acids, phenylethanoid, iridoid, and lignan glycosides, and flavonoids. The *L. nubica* extract profile was dominated by the phenylethanoid glycoside verbascoside. All annotated compounds are reported for the first time in the species. The extract actively scavenged DPPH and ABTS radicals (38.8 ± 0.1 and 36.8 ± 0.4 mg TE/g) and showed high CUPRAC (71.1 ± 1.1 mg TE/g) and moderate FRAP (44.9 ± 2.6 mg TE/g) reducing power. The *L. nubica* extract exhibited high inhibition towards acetylcholinesterase (2.23 ± 0.02 mg GALAE/g), butyrylcholinesterase (2.38 ± 0.04 mg GALAE/g), and tyrosinase (60.7 ± 0.6 mg KAE/g). The obtained results highlight *L. nubica* extract as a rich source of phenylethanoid glycosides and flavonoids with significant bioactivity and shed light into the phytochemical composition and pharmacological potential of the plant.

## 1. Introduction

The *Leucas* R. B. genus (Lamiaceaea family) includes herbs or subshrubs distributed throughout African and Asian tropical and temperate countries [1]. *L. nubica* Benth. is an annual herb characterized by cymes with white or pale-colored flowers and trigonous-oblong nutlets [2]. The species, commonly known as “Mayoub”, is native to east and northeast tropical Africa. The whole plant is renowned for the treatment of jaundice in the traditional medicine of eastern Sudan [3].

In a previous study of *L. nubica* whole-plant extracts, the total phenolic content ranged between 0.216 gallic acid/g (dichloromethane extract) and 1.015 mg gallic acid/g (methanol extract), while total flavonoids varied from 0.400 mg quercetin/g to 0.580 mg quercetin/g, respectively [3]. In the antioxidant potential assessment assays, methanol and ethyl acetate extracts scavenged DPPH radicals (47%) and DMPD (48%), respectively, whereas reducing power was estimated at 1.117 (absorbance in FRAP) and 0.361 (absorbance in PRAP), respectively. Metal chelating capacity reached 48% (ethyl acetate extract). *L. nubica* extracts (at 10 mg/mL) have been shown to not be cytotoxic towards human CCRF-CEM leukemia cells. On the other hand, in Africa and Asia, *Leucas* species, including *L. aspera* (Willd.) Link., *L. ciliata* Benth. and *L. lavandulifolia* Sm., are used in an ethnopharmacological approach as anti-inflammatory, antipyretic, analgesic and anti-bacterial agents [4,5,6]. Indeed, lignans, flavonoids, and isopimarane and spiro-labdane diterpenoids hold significance for their anti-inflammatory and antioxidant activity.

To the best of our knowledge, there is no data on the phytochemical composition of *L. nubica*. Taken together, the cited studies generated further interest in the species and prompted us to undertake in-depth profiling of secondary metabolites in *L. nubica* aerial parts by means of ultra-high-performance liquid chromatography coupled with hybrid quadrupole–Orbitrap high-resolution mass spectrometry. This study was combined with an assessment of antioxidant activity and enzyme inhibitory potential towards key targets in therapies, including cholinesterases, α-amylase, α-glucosidase, and tyrosinase.

## 2. Results and Discussion

### 2.1. LC-HRMS Profiling of Leucas nubica Extract

*L. nubica* metabolite profiling was carried out according to the Çiçek et al., 2024 approach in order to be of maximum scientific relevance [7]. Identification confidence levels were as follows: confirmed structure including confirmed stereochemistry (A2); confirmed structure except for one or more stereochemical aspects (B); tentative identification matched with a standard compound, match of at least t_R_, MS and MS/MS with an actual authentic standard analyzed in parallel, preferably supported by other online data (C); tentative identification based on libraries, model compounds etc. (D), relatively reliable evidence (D1); and relatively poor evidence (D2).

Based on the retention times, MS and MS/MS accurate masses, fragmentation patterns in MS/MS spectra, relative ion abundances, and comparison of retention times with reference standards and literature data, 78 metabolites were identified or tentatively annotated in *L. nubica* extract.

#### 2.1.1. Sugar Acids, Saccharides, Carboxylic, and Phenolic Acids

Compounds 1–4 were tentatively annotated as xylonic acid, hexose, gluconic acid, and asystoside, while 5–8 were annotated as malic, citric/isocitric, oxaloglutaric and quinic acid, respectively. The dereplication was based on comparison of MS and MS/MS spectra with literature data (Table 1 and Table S1) [8,9]. Five hydroxybenzoic acids (11, 16, 21, 22, and 27), five hydroxycinnamic acids (9, 13, 17, 20, and 23), their glycosides (10, 14, 15, 18, 19, 24, 25, and 26), and vanillyl alcohol (12) were identified based on the comparison with reference standards and literature data in the assayed extract (Table 1 and Table S1) (Figures S1–S7) [8,9].

#### 2.1.2. Phenylethanoid Glycosides Annotation

**Figure 1:**
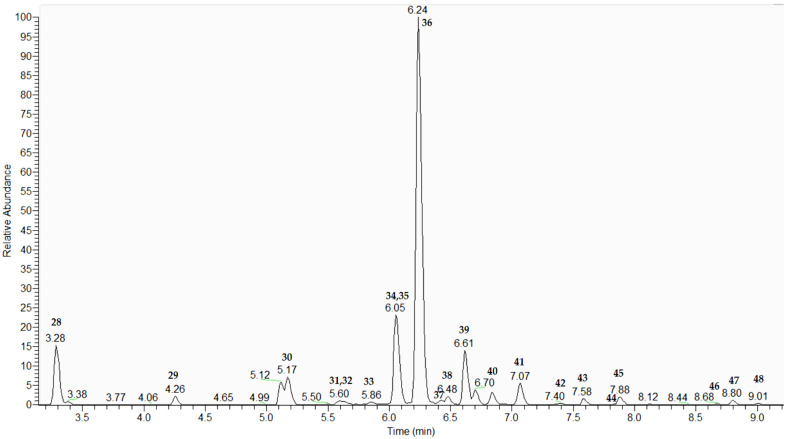
Extracted ion chromatogram (EIC) of phenylethanoids. EIC was conducted with a mass tolerance of 5 ppm (for numbers and fragmentation patterns, see Table 1).

Phenylethanoid glycosides are a class of secondary metabolites usually presented in Lamiaceae species. The typical fragmentation pattern revealed losses of 162.05, 146.05, 132.02, and 162.03 Da, corresponding to hexosyl (Hex), deoxyhexosyl (dHex), pentosyl, and caffeoyl residues, respectively. Detailed discussion on the MS/MS fragmentation was previously described [9]. Among all detected compounds, 21 were classified as phenylethanoid glycosides. The extracted ion chromatogram is presented in Figure 1.

**Figure 2:**
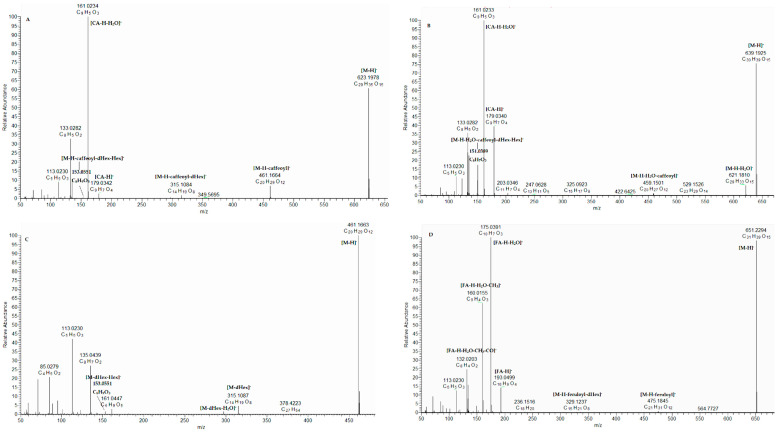
(-) ESI-MS/MS spectrum of verbascoside (36) (**A**), hydroxyverbascoside (30) (**B**), decaffeoyl-verbascoside (28) (**C**), and martynoside (45) (**D**).

Compounds 36 and 39 with deprotonated molecular ions at *m*/*z* 623.198 shared the typical fragmentation pattern for verbascoside and isoverbascoside, with fragments at *m*/*z* 461.166 [M−H-caffeoyl]^−^ and 315.108 [M−H-caffeoyl-dHex]^−^, together with ions, corresponding to caffeic acid (CA) at *m*/*z* 179.034 [CA-H]^−^, 161.023 [CA-H-H_2_O]^−^, and 135.044 [CA-H-CO_2_]^−^ (Table S1, Figure 2A). Verbascoside (36) was unambiguously identified by comparison with the reference standard. Compounds 30/33 with [M-H]^−^ at *m*/*z* 639.193 were annotated as hydroxyverbascoside and its isomer, as they showed a similar fragmentation pattern to verbascoside, with the additional characteristic ion at *m*/*z* 151.039 corresponding to hydroxyvinyl-benzene-diol residue (Table S1, Figure 2B). In the same way, compound 32 [M-H]^−^ at *m*/*z* 667.188 was related to carboxyverbascoside based on an additional neutral loss of CO_2_ at *m*/*z* 623.196 (Figure S8). Compound 28 ([M-H]^−^ at *m*/*z* 461.166) shared the same fragmentation pattern except for the caffeoyl residue. Accordingly, 28 was assigned as decaffeoyl-verbascoside (Table 1, Figure 2C). Compounds 31/35 with [M-H]^−^ at *m*/*z* 785.251 differed from verbascoside with an additional hexosyl moiety and were ascribed to echinacoside and its isomer (Table 1, Figure S9). MS/MS spectrum of compound 45 afforded fragment ions at *m*/*z* 475.185 and 329.124, corresponding to the consecutive losses of feruloyl and deoxyhexosyl residues, and a base peak was consistent with dehydrated ferulic acid (FA). Thus, 45 was dereplicated as martynoside [10] (Table 1) (Figure 2D). Compounds 41 and 43 gave similar fragmentation pathways to martynoside, corroborated by the fragment ion at *m*/*z* 153.055 [M-H-feruloyl-deoxyhex-hex]^−^, corresponding to the hydroxytyrosol moiety. Thus, the compounds were dereplicated as leucoseptoside A and its isomer (Figure S10) [11]. Compounds 40 ([M-H]^−^ at *m*/*z* 769.256) and 42 ([M-H]^−^ at *m*/*z* 783.271) possessed an additional pentose in comparison with leucoseptoside and martynoside, respectively. Accordingly, they were ascribed to alyssonoside (Figure S11) [12] and leontoside B (Figure S12), respectively. Owing to the fact that compound 44 had a supplementary acetyl group compared to verbascoside, it was related to acetylvarbascoside/acetylacteoside (Figure S13). Similarly, 46 and 48 were ascribed to acetylmarthinoside and its isomer [13] (Table 1, Figure S14). Compound 29 [M-H]^−^ at *m*/*z* 475.182 yielded indicative fragment ions at *m*/*z* 167.070 [C_9_H_11_O_3_]^−^ and 134.036 [C_9_H_11_O_3_-H_2_O-CH_3_]^−^, corresponding to methoxyphenyl-hydroxyethyl residue, and 29 was annotated as darendoside B [14] (Figure S15). Compounds 34 and 38 were dereplicated as forsythoside B/samioside/lavandulifolioside based on the consecutive loss of caffeoyl residue, pentose, and deoxyhexose [10] (Figure S16). The MS/MS spectrum of 37 showed an indicative ion at *m*/*z* 323.077 [M-H-C_8_H_10_O_3_]^−^ and was annotated as calceolarioside [15] (Figure S17). Compound 47 [M-H]^−^ at *m*/*z* 591.208 differed from verbascoside by the absence of OH groups in the hydroxytyrosol residue and was related to jionoside C (Figure S18).

#### 2.1.3. Iridoid and Lignan Glycosides Annotation

**Figure 3:**
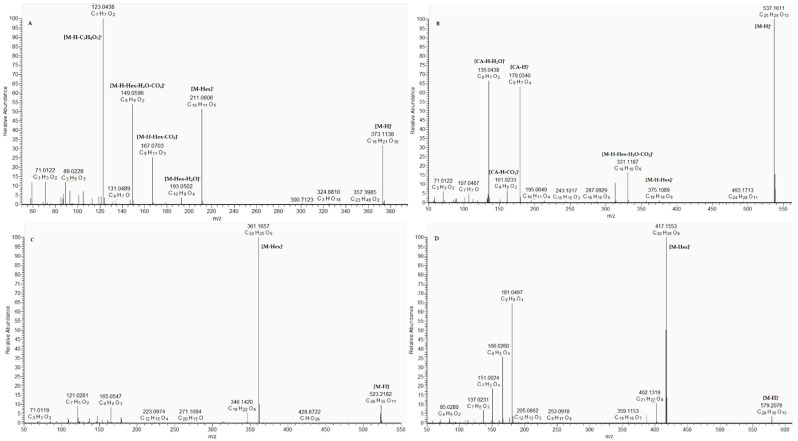
(-) ESI-MS/MS spectrum of geniposidic acid (49) (**A**), caffeoyl mussaenosidic acid (52) (**B**), secoisolariciresinol O-hexoside (51) (**C**), syringaresinol O-hexosides (53) (**D**).

The extracted ion chromatogram of the annotated iridoid and lignan glycosides in negative ion mode is depicted in Figure S19. Compound 49 [M-H]^−^ at *m*/*z* 373.114 was dereplicated as geniposidic acid, based on the indicative ions at *m*/*z* 211.061 [M-H-Hex]^−^, 193.050 [M-H-Hex-H_2_O]^−^, 167.070 [M-H-Hex-CO_2_]^−^, 149.060 [M-H-Hex-H_2_O-CO_2_]^−^, and 123.044 [M-H-C_3_H_4_O_3_]^−^ [16] (Figure 3A) (Table S1). Similarly, but with an additional OH group, compound 50 was related to monotropein [17] (Figure S20). Compound 52 [M-H]^−^ at *m*/*z* 537.161 gave fragments at *m*/*z* 375.109 [M-H-Hex]^−^, 357.100 [M-H-Hex-H_2_O]^−^, 331.119 357.100 [M-H-Hex-H_2_O-CO_2_]^−^, and 313.108 [M-H-Hex-2H_2_O-CO_2_]^−^, together with ions at *m*/*z* 179.030 [CA-H]-, 161.023 [CA-H-CO_2_]^−^, and 135.044 [CA-H-H_2_O]^−^. Thus, 52 was annotated as caffeoyl mussaenosidic acid [18] (Figure 3B). Based on comparison with literature data, two lignans 51 and 53 were dereplicated as secoisolariciresinol and syringaresinol *O*-hexosides, respectively [19] (Figure 3C,D) (Table S1).

#### 2.1.4. Flavonoids Annotation

**Figure 4:**
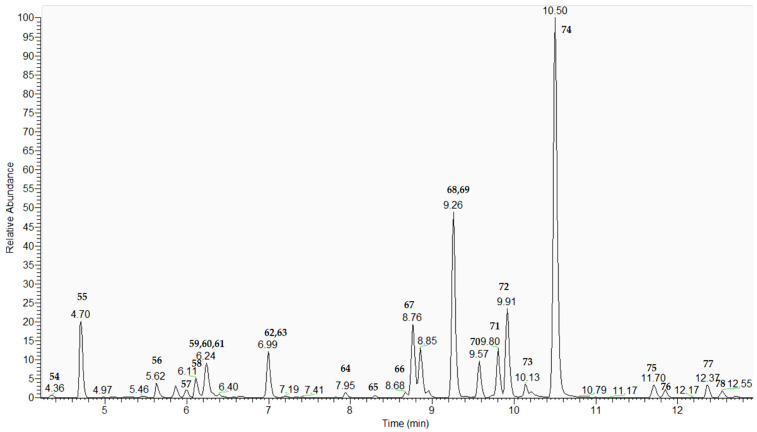
Extracted ion chromatogram (EIC) of flavonoids. EIC was conducted with a mass tolerance of 5 ppm (for numbers and fragmentation patterns, see Table 1).

Twenty-five flavonoids belonging to the class of flavones and flavanones were annotated in *L. nubica* extract (Figure 4). They contain apigenin, luteolin, cirsilol, chrysoeriol, nepetin, diosmetin, velutin, naringenin, eriodictyol, an aglycone moiety and their mono-, di-, and coumaroylglycosides (Table 1 and Table S1). The flavonoids dereplication strategy was previously detailed discussed [20,21].

Based on the comparison with reference standards, 57, 61, 63, 64, 66, 67, 72, and 74 were unambiguously identified as isovitexin, luteolin 7-*O*-glucoside, apigenin 7-*O*-glucoside, nepetin, eriodictyol, luteolin, apigenin, and cirsiliol, respectively. The MS/MS spectra of the aforementioned compounds are presented in Figures S21–S28. Compounds 68 and 70 gave a precursor ion at [M-H]^−^ at *m*/*z* 577.135 together with fragment ions at *m*/*z* 431.098, [M-H-146.036]^−^, and 145.028 (C_9_H_5_O_2_), corresponding to the loss of coumaroyl residue. The base peak at *m*/*z* 269.046 [M-H-coumaroyl-Hex]^−^ and fragments at *m*/*z* 151.002 (^1,3^A^−^) and 117.033 (^1,3^B^−^) were due to the aglycon apigenin (Table S1).

**Figure 5:**
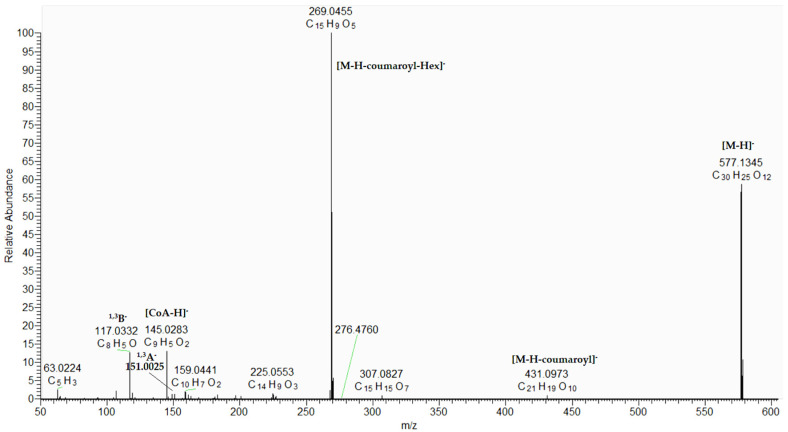
(-) ESI-MS/MS spectrum of apigenin 7-*O*-coumaroylhaxoside (68).

Thus, 68/70 were annotated as apigenin 7-*O*-coumaroylhexoside and its isomer (Table S1, Figure 5). Similarly, 77/78 were related to apigenin 7-*O*-dicoumaroylhexoside (Figure S29), while 71/73 were annotated as naringenin 7-*O*-coumaroylhexoside and its isomer [22]. (Table 1, Figure S30).

In general, in the studied *L. indica* extract, 15 compounds were identified at level B, 1 at level C, 41 at level D1, and 21 at level D2 (Table 1).

### 2.2. Determination of Total Phenolic and Flavonoid Contents in L. nubica Extract

In this study, we determined the total phenolic and flavonoid contents of *L. nubica* extract, and the results are presented in Table 2. The levels of total phenolics and flavonoids were found to be 22.1 mg gallic acid equivalent per gram (GAE/g) and 4.37 mg rutin equivalent per gram (RE/g), respectively. Different results for the total bioactive compounds have been reported in the literature. Previously, Adam et al. (2018) detected total phenolic and flavonoid contents of 0.216–1.015 mg/g and 0.400–0.580 mg/g in three *L. nubica* extracts, respectively [3]. Another study by Ali et al. (2013) reported these values for the methanol extract of *L. aspera* as 131.15 mg GAE/g and 135.85 mg QE/g, respectively [23]. Furthermore, the total phenolic and flavonoid contents were found to be 164.96 mg GAE/g and 36.95 mg RE/g, respectively, in the methanol extract of *L. cephalotes* [24]. Chetia and Saikia (2020) investigated the total phenolic and flavonoid content of extracts from various parts of *L. aspera*, reporting that polar extracts were particularly rich in these bioactive compounds [25]. Hakim et al. (2021) also found that the total phenolic content was 484.88 mg GAE/100 g of fresh material in the methanol extract of *L. aspera* [26]. The different levels obtained in the reported results can be explained by geographical and climatic differences, as well as by the units of the used measurement.

### 2.3. Elucidation of Antioxidant Potential of L. nubica Extract

Plant secondary metabolites such as phenylethanoid and flavonoid glycosides are among the best-known and most active antioxidant molecules [27,28]. The current study examined the antioxidant properties of *L. nubica* extract using various chemical assays, including radical scavenging, reducing power and metal chelating. The results are presented in Table 2. DPPH and ABTS ^(•+)^ radicals are the most common methods used to detect the hydrogen donation ability of antioxidant compounds. In the present study, the tested extract exhibited scavenging ability against both radicals (DPPH: 38.3 mg TE/g; ABTS: 36.8 mg TE/g). Reducing power is a significant parameter in antioxidant evaluation and is related to the ability to donate electrons. CUPRAC and FRAP assays were used for the measurement of reducing power. The tested extract exhibited reduction potential (CUPRAC: 71.1 mg TE/g; FRAP: 44.9 mg TE/g). Similar to CUPRAC and FRAP assays, the phosphomolybdenum (PBD) assay involves the reduction of Mo(VI) to Mo(V) by antioxidants under acidic conditions. The ability of the tested extract in the PBD assay was 1.33 mmol TE/g. Regarding metal chelation, the chelation of ferrous ions can control the production of hydroxyl radicals in the Fenton reaction. As shown in Table 2, the tested extract exhibited metal chelating ability, with 8.91 mg EDTA/g.

Several researchers have reported the antioxidant properties of *Leucas* species, including *L. nubica*. For instance, Adam et al. (2018) studied three *L. nubica* extracts and found that the methanol extract exhibited the greatest DPPH scavenging ability at a concentration of 1 mg/mL [3]. Sakthidhasan et al. (2022) also investigated the antioxidant properties of *L. lavandulifolia*, finding that its methanol extract exhibited the best DPPH scavenging ability (IC_50_: 3.91 µg/mL), as well as ferric reducing ability [29]. Chew et al. (2012) evaluated the radical scavenging ability of different parts of *L. aspera* and found that the root displayed the best DPPH scavenging ability at a concentration of 2 mg/mL [30]. Aryal et al. (2019) revealed that the methanol extract of *L. cephalotes* exhibited remarkable DPPH scavenging and ferric reducing effects [24]. Gangadharan and Benny (2021) examined the different extracts of *L. aspera* for antioxidant properties, finding that the methanol extract possessed the highest radical scavenging and ferric reducing effects [31]. Taken together, we conclude that a polar solvent can be useful for obtaining extracts from the *Leucas* genus.

*L. nubica* contains verbascoside (VB), which has been reported to exert powerful antioxidant activity in several assays (TEAC, ORAC, HORAC, FRAP, CUPRAC) [32]. Furthermore, VB actively scavenges superoxide anion, hydrogen peroxide, nitic oxide and peroxy-nitrite radicals. Indeed, VB manifested a greater direct ROS scavenging capacity in comparison with Trolox. Thus, IC_50_ values of 7.6 μM for VB and 24.2 μM for Trolox were evaluated in the DPPH assay, while IC_50_ values of 731 μM and 1205 μM were determined for VB and Trolox, respectively, in the superoxide anion (^•^O_2_^−^) assay [33]. The same trends have been reported in a comparative study of VB and ascorbinic acid, where in DPPH^•^, ^•^O_2_^−^ and hydroxyl radical (^•^OH) assays, IC_50_ values of 58.1, 24.4 and 357 μM were estimated for VB and IC_50_ values of 284.9, 66.1 and 1031 μM were found for ascorbinic acid [34]. Owing to the fact that hydroxyl radicals are extremely harmful, the scavenging capacity of hydroxyl radicals appears to be basically associated with the prevention of lipid peroxydation. Furthermore, VB-rich extract from *Scutellaria laterifoli* (485 mg/100 g dw) showed high metal chelating capacity [35]. It is worth noting that in models of H_2_O_2_-stressed HepG2 and SH-SY5Y cells, pre-treatment with VB significantly reduced intracellular ROS levels with respect to the stressed control [34]. Taken together, these results are in line with the neuroprotective effects established for nutraceutical products rich in VB from *Olea europaea* and *Hybiscus sabdarifa* in a model of oxidative stress injury in human neuroblastoma SH-SY5Y [36]. The capacity of VB to scavenge free radicals could also be related to its chemopreventive capacity in cell culture under oxidative stress, as shown in the aforementioned studies. It has been established that VB passes the blood–brain barrier, preventing ROS accumulation and preserving the antioxidant system [37].

In addition to evoking free radical scavenging, the antioxidant mechanism of VB was ascribed to the gene transcription of catalase, glutathione peroxidase and other antioxidant enzymes through the nuclear factor erythroid 2-related factor (Nrf2) pathway, which is the primary transcriptional regulator of the cellular antioxidant response [38].

In addition, verbascoside, cirsiliol, and apigenin 7-O-glucoside being among predominant flavonoids in the extract could also contribute to the antioxidant activity of the extract. It have been proven that these flavonoids neutralize reactive oxygen species and protect cells from oxidative damage by influencing antioxidant pathways (like Nrf-2/NF-κB) [39,40].

### 2.4. Elucidation of Enzyme Inhibitory Potential of L. nubica Extract

To provide new raw material for safer enzyme inhibitors, we studied the enzyme inhibitory properties of the *L. nubica* extract against cholinesterase, amylase, glucosidase, and tyrosinase. The results are presented in Table 2. The inhibitory effects on AChE and BChE were 2.23 mg GALAE/g and 2.38 mg GALAE/g, respectively. The inhibition values for amylase and glucosidase were 0.41 mmol ACAE/g and 1.62 mmol ACAE/g, respectively. Regarding tyrosinase inhibition, the extract showed an IC_50_ of 60.73 mg KAE/g. There are few reports on the enzyme inhibitory properties of *Leucas* species. In a study by Meera et al. (2017), a protein isolated from *L. aspera* exhibited potent amylase inhibition (90%) [41]. In another study by Verma et al. (2017), the amylase inhibitory effects of different parts of *L. cephalotes* were examined, and the fruits and leaves exhibited potent inhibition, with values exceeding 70% [41]. The n-hexane extract of *L. cephalotes* displayed an inhibitory effect on AChE with a value of 78.7%, as reported by Shahwar et al. (2012) [42]. In another study by Fatima et al. (2008), a new sterol (leucisterol) was isolated from *L. urticifolia*, and it exhibited a significant inhibitory effect on butyrylcholinesterase [43]. As can be seen in Table 2, the tested extract contained compounds, including apigenin, luteolin, caffeic and chlorogenic acid, which are already known to be enzyme inhibitors [44,45]. Thus, the methanol extract of *L. nubica* can be considered a valuable source of natural enzyme inhibitors.

It was reported that verbascoside possessed significant enzyme inhibitory activity towards acetyl cholinesterase and butyrylcholinesterase, with IC_50_ values of 19.9 and 35.0 μM (IC50 of bereberin was 0.09 μM) [46]. Verbascoside has promising β-secretase inhibitory activity, with an IC_50_ value of 6.3 nM alongside commercial olive fruit extracts (up to 20 μg) [47]. Interestingly, the cholinesterase inhibitory activity of VB was not detected in the aforementioned study. In another work, VB was tested in a wide concentration range (up to 350 μM), but the AChE inhibition did not exceed 18% (https://doi.org/10.3390/antiox9121207). On the other hand, VB-rich plant methanol extracts from *Veronica teucrium* and *V. jacquinii* at 50 mg/mL exhibited effective AChE activity (up to 35% enzyme inhibition), which pointed out the synergistic role of bioactive compounds in the extracts [48].

The data on the enzyme inhibitory activity of VB against tyrosinase are contradictory. It has been reported to have a dose-dependent activation of the enzyme, which was not pronounced in the highest concentration [34]. On the other hand, it was not detected in the study of olive fruit extracts and their main constituents [47].

Apigenin is a flavonoid with notable inhibitory activity against AChE, primarily mediated by its ability to form hydrogen bonds with key amino acid residues at the enzyme’s active site, including Ser200 and His440. Hydrophobic interactions further stabilize apigenin within the active site, resulting in a dose-dependent reduction in AChE activity with an IC50 value of 40.7 µM [44]. Recently, O-glucosides of apigenin and luteolin demonstrated weak tyrosinase and butyrylcholine esterase inhibition, while luteolin-7-O-glucoside showed a more significant inhibitory effect against acetylcholinesterase (65 ± 2%) [49].

## 3. Materials and Methods

### 3.1. Plant Material

*Leucas nubica* aerial parts were collected from Erkowit city (18°42′0″ N, 37°0′0″ E), eastern Sudan, during the full flowering stage, in December, 2024. The plant material was identified by Prof. Maha Kordofani (taxonomist). A voucher specimen was deposited at the Herbarium, KHU (No. LN/S18). The collected plant material was dried at room temperature (20–22 °C) and 50% relative humidity. The aerial parts were dried until a constant weight of the plant material.

### 3.2. Sample Extraction

Air-dried powdered aerial parts (20 g) were extracted with 50% MeOH (1:30 *w*/*v*) by ultrasound (100 kHz, ultra-sound bath Biobase UC-20C, Jinan, China) for 15 min, three times, at room temperature. The methanol was then evaporated in vacuo, and water residue was lyophilized (lyophilizer Biobase BK-FD10P) to yield crude extract of 2.6 g. Then, the lyophilized extract was dissolved in 50% methanol (0.1 mg/mL), filtered through a 0.45 μm syringe filter and subjected to UHPLC-HRMS analyses. The same extract was used for further biological tests.

### 3.3. Chemicals

Acetonitrile (for LC–MS), formic acid (for LC–MS) and methanol (for HPLC) were purchased from Honeywell (Charlotte, CA, USA). The reference standards were obtained from Extrasynthese (Genay, France) (for protocatechuic, o-coumaric, caffeic, p-hydroxyphenylacetic acid, gentisic acid, isovitexin, luteolin 7-*O*-glucoside, eriodictyol, apigenin, nepetin) and Phytolab (Vesten-bergsgreuth, Bavaria, Germany) (chlorogenic acid, verbascoside).

### 3.4. UHPLC-HRMS

The UHPLC-HRMS analyses were performed as previously described [50] on a Q Exactive Plus mass spectrometer (ThermoFisher Scientific, Inc., Waltham, MA, USA) with a heated electrospray ionization (HESI-II) probe in negative and positive ion modes within the *m*/*z* range from 150 to 1500. Chromatographic separation was achieved on a reversed-phase column C18 (1.8 µm, 2.1 × 100 mm), and the temperature was 40 °C. The mobile phase consisted of 0.1% formic acid in water (A) and 0.1% formic acid in acetonitrile (B), the run time was 33 min, and the flow rate and other chromatographic conditions were previously described [50]. The Xcalibur 4.2 software (ThermoScientific, Waltham, MA, USA) was used for data processing.

### 3.5. Assay for Total Phenolic and Flavonoid Contents

Total phenolic and flavonoid content quantification was carried out according to the methods previously described [51]. Gallic acid (GA) and rutin (RE) served as standards in the assays, and the outcomes were reported as gallic acid equivalents (GAE) and rutin equivalents. The experimental details are given in the Supplementary Materials.

### 3.6. Assays for In Vitro Antioxidant Capacity

The antioxidant activity of the studied extract was evaluated [52]. The DPPH, ABTS radical scavenging, CUPRAC, and FRAP test results were presented as milligrams of Trolox equivalents (TE) per gram of extract. The antioxidant potential determined by the phosphomolybdenum (PBD) assay was measured in millimoles of Trolox equivalents (TE) per gram of extract, and the metal chelating activity (MCA) was conveyed as milligrams of disodium edetate equivalents (EDTAE) per gram of extract. The experimental details are given in the Supplementary Materials.

### 3.7. Inhibitory Effects Against Some Key Enzymes

Enzyme inhibition experiments on the samples were conducted following established protocols [52]. Amylase and glucosidase inhibition were quantified in mmol acarbose equivalents (ACAE) per gram of extract, while acetylcholinesterase (AChE) and butyrylcholinesterase (BChE) inhibition were expressed in milligrams of galanthamine equivalents (GALAE) per gram of extract. Tyrosinase inhibition was measured in milligrams of kojic acid equivalents (KAE) per gram of extract. The experimental details are given in the Supplementary Materials.

## 4. Conclusions

This study is the first attempt to delineate the phytochemical profiling of the unexplored plant *L. nubica*. In summary, we demonstrated that the methanol–aqueous extract from the aerial parts is a rich source of phenylethanoid glycosides alongside iridoids, flavonoids and lignans. Based on UHPLC-HRMS analysis, verbascoside was among the dominant secondary metabolites together with the flavonoids cirsiliol and apigenin glycosides, correlating closely with the antioxidant effects of the plant extract. The occurrence of verbascoside supports the observed inhibitory activity towards acetyl- and butyrylcholinesterase. Furthermore, an inhibitory activity was proven against the key enzyme in melanin biosynthesis, tyrosinase. In the context of carbohydrate metabolism, the studied extract possesses promising a glucosidase inhibitory activity along with moderate α-amylase inhibition. Indeed, our findings suggest *L. nubica* as a hopeful candidate for the prevention of conditions associated with oxidative stress and neurodegeneration. Thus, the plant extract would need to be explored in in vitro/in vivo models in the context of neuroprotection.

## Figures and Tables

**Table 1:** LC-HRMS metabolite profiling of *Leucas nubica* extract.

No.	Identified/Tentatively Annotated Compound	Molecular Formula	Exact Mass [M-H]^−^	t_R_ (min)	Level of Confidence [7]
**Sugar** **acids and saccharides**					
**1.**	xylonic acid	C_5_H_10_O_6_	165.0405	0.73	D1
**2.**	hexose	C_6_H_12_O_6_	179.0561	0.79	D1
**3.**	gluconic acid	C_6_H_12_O_7_	195.0510	0.74	D1
**4.**	asystoside/ebracteatoside B/lunaroside	C_25_H_44_O_15_	583.2607	7.12	D2
**Carboxylic acids**					
**5.**	malic acid ^a^	C_4_H_6_O_5_	133.0142	0.81	B
**6.**	citric /isocitric acid	C_6_H_8_O_7_	191.0197	1.11	D1
**7.**	oxaloglutaric acid	C_7_H_8_O_7_	203.0197	1.13	D1
**8.**	quinic acid	C_7_H_12_O_6_	191.0561	2.34	D1
**Hydroxybenzoic, hydroxycinnamic, acylquinic acids, and their derivatives**					
**9.**	salvianic acid A	C_9_H_10_O_5_	197.0455	2.63	D1
**10.**	dihydrocaffeic acid O-hexoside	C_15_H_20_O_9_	343.1035	2.71	D2
**11.**	protocatechuic acid ^a^	C_7_H_6_O_4_	153.0193	2.95	B
**12.**	vanillyl alcochol	C_8_H_10_O_3_	153.0557	3.07	D1
**13.**	chlorogenic acid ^a^	C_16_H_18_O_9_	353.0878	3.19	B
**14.**	peiioside B	C_25_H_38_O_16_	593.2087	3.26	D2
**15.**	caffeic acid *O*-rutinoside (swertiamacroside)	C_21_H_28_O_13_	487.1457	3.50	D1
**16.**	dihydroxybenzoic acid	C_7_H_6_O_4_	153.0193	4.14	D2
**17.**	*p*-coumaric acid ^a^	C_9_H_8_O_3_	163.0401	4.19	B
**18.**	*p*-coumaric acid *O*-hexoside	C_15_H_18_O_8_	325.0928	4.20	D1
**19.**	ferulic acid *O*-hexosyl-deoxyhexoside	C_22_H_30_O_13_	501.1614	4.57	D1
**20.**	*p*-hydroxyphenyl acetic acid ^a^	C_8_H_8_O_3_	151.0400	4.59	B
**21.**	caffeic acid ^a^	C_9_H_8_O_4_	179.0350	4.61	B
**22.**	gentisic acid ^a^	C_7_H_6_O_4_	153.0193	4.91	B
**23.**	*o*-coumaric acid ^a^	C_9_H_8_O_3_	163.0401	5.72	B
**24.**	dicaffeoylhexose	C_24_H_24_O_12_	503.1195	6.38	D2
**25.**	syringalide A-deoxyhexoside	C_29_H_36_O_14_	607.2032	6.83	D2
**26.**	dicaffeoylhexose	C_24_H_24_O_12_	503.1195	7.03	D2
**27.**	hydroxybenzoic acid	C_7_H_6_O_3_	137.0244	7.98	D2
**Phenylethanoid glycosides**					
**28.**	decaffeoyl aceteoside/ decaffeoyl verbasoside	C_20_H_30_O_12_	461.1664	3.28	D1
**29.**	darendoside B	C_21_H_32_O_12_	475.1821	4.26	D2
**30.**	hydroxyverbascoside	C_29_H_36_O_16_	639.1931	5.17	D1
**31.**	echinacoside	C_35_H_46_O_20_	785.2510	5.60	D1
**32.**	carboxyverbascoside	C_30_H_36_O_17_	667.1880	5.65	E
**33.**	hydroxyverbascoside isomer	C_29_H_36_O_16_	639.1931	5.86	D1
**34.**	forsythoside B/samioside/lavandulifolioside	C_34_H_44_O_19_	755.2404	6.05	D1
**35.**	echinacoside isomer	C_35_H_46_O_20_	785.2510	6.08	D1
**36.**	verbascoside ^a^	C_29_H_36_O_15_	623.1981	6.24	C
**37.**	calceolarioside	C_23_H_26_O_11_	477.1402	6.43	D1
**38.**	forsythoside B/samioside/lavandulifolioside	C_34_H_44_O_19_	755.2404	6.48	D1
**39.**	isoverbascoside	C_29_H_36_O_15_	623.1981	6.61	D1
**40.**	alyssonoside	C_35_H_46_O_19_	769.2561	6.83	D1
**41.**	leucoseptoside A	C_30_H_38_O_15_	637.2138	7.07	D1
**42.**	leontoside B/stachyoside D	C_36_H_48_O_19_	783.2717	7.40	D1
**43.**	leucoseptoside A isomer	C_30_H_38_O_15_	637.2138	7.58	D1
**44.**	acetylverbascoside	C_31_H_38_O_16_	665.2087	7.84	D2
**45.**	martynoside	C_31_H_40_O_15_	651.2294	7.88	D1
**46.**	acetylmartynoside	C_33_H_42_O_16_	693.2400	8.68	D2
**47.**	jionoside C	C_29_H_36_O_13_	591.2083	8.80	D2
**48.**	acetylmartynoside isomer	C_33_H_42_O_16_	693.2400	9.01	D2
**Iridoid and lignan glycosides**					
**49.**	geniposidic acid	C_16_H_22_O_10_	373.1140	2.70	D2
**50.**	monotropein	C_16_H_22_O_11_	389.1089	3.06	D2
**51.**	secoisolariciresinol O-hexoside	C_26_H_36_O_11_	523.2185	5.71	D1
**52.**	caffeoyl-mussaenosidic acid	C_25_H_30_O_13_	537.1614	6.90	D2
**53.**	syringaresinol O-hexoside	C_28_H_36_O_13_	579.2083	6.91	D1
**Flavonoids**					
**54.**	naringenin 6,8-*C*-dihexoside	C_27_H_32_O_15_	595.1668	4.36	D1
**55.**	apigenin 6,8-*C*-hexosyl hexoside	C_27_H_30_O_15_	593.1512	4.70	D1
**56.**	luteolin 7-*O*-dihexoside	C_27_H_30_O_16_	609.1461	5.62	D2
**57.**	isovitexin ^a^	C_21_H_20_O_10_	431.0984	5.99	B
**58.**	apigenin-*O*-deoxyhexosylhexoside	C_27_H_28_O_16_	607.1305	6.11	D1
**59.**	apigenin *O*-hexosyl-hexoside	C_27_H_30_O_15_	593.1512	6.24	D1
**60.**	luteolin 7-*O*-hexuronide	C_27_H_30_O_16_	461.0725	6.23	D1
**61.**	luteolin 7-*O*-glucoside ^a^	C_21_H_20_O_11_	447.0933	6.24	B
**62.**	luteolin 4′-*O*-hexoside	C_21_H_20_O_10_	447.0933	6.99	D1
**63.**	apigenin 7-*O*-glucoside ^a^	C_21_H_20_O_10_	431.0984	7.00	B
**64.**	nepetin ^a^	C_16_H_12_O_7_	315.0510	7.95	B
**65.**	cirsiliol-*O*-hexoside	C_23_H_24_O_12_	491.1195	8.29	D1
**66.**	eriodictyol ^a^	C_15_H_12_O_6_	287.0561	8.68	B
**67.**	luteolin ^a^	C_15_H_10_O_6_	285.0405	8.76	B
**68.**	apigenin 7-*O*-coumaroylhexoside	C_30_H_26_O_12_	577.1351	9.26	D1
**69.**	chrysoeriol/diosmetin O-hexoside	C_22_H_22_O_11_	461.1089	9.27	D1
**70.**	apigenin 7-*O*-coumaroylhexoside isomer	C_30_H_26_O_12_	577.1351	9.57	D1
**71.**	naringenin 7-*O*-coumaroylhexoside	C_30_H_28_O_12_	579.1508	9.80	D1
**72.**	apigenin ^a^	C_15_H_10_O_5_	269.0455	9.91	B
**73.**	naringenin 7-O-coumaroylhexoside isomer	C_30_H_28_O_12_	579.1508	10.13	D1
**74.**	cirsiliol ^a^	C_17_H_14_O_7_	329.0667	10.50	B
**75.**	diosmetin	C_16_H_12_O_6_	299.0561	11.70	D1
**76.**	velutin	C_17_H_14_O_6_	313.0718	11.86	D1
**77.**	apigenin 7-*O*-dicoumaroyl-*O*-hexoside	C_39_H_32_O_14_	723.1719	12.36	D2
**78.**	apigenin 7-*O*-dicoumaroyl-*O*-hexoside isomer	C_39_H_32_O_14_	723.1719	12.55	D2

[M-H]^−^: deprotonated molecular ion; exact mass: calculated mass of an ion whose elemental formula, isotopic composition and charge state are known, i.e., the theoretical mass; t_R_: retention time; Δ ppm: delta parts per million—a measurement of the mass accuracy, or the difference between an experimentally measured mass and its theoretically calculated mass; confidence level: B: confirmed structure except for one or more stereochemical aspects; C: tentative identification matched with a standard compound, match of at least t_R_, MS and MS/MS with an actual authentic standard analyzed in parallel, preferably supported by other online data; D: Tentative identification based on libraries, model compounds etc.; D1: relatively reliable evidence; D2: relatively poor evidence; E: tentative candidate or tentative identification of metabolite class [7]; ^a^: compared to reference standard.

**Table 2:** Total phenolic and flavonoid contents and antioxidant and enzyme inhibitory properties of *L. nubica* extract.

*Total Bioactive Compounds*	
Total phenolic content (mg GAE/g)	22.1 ± 0.1
Total flavonoid content (mg RE/g)	4.37 ± 0.04
***Antioxidant activity***	
DPPH scavenging ability (mg TE/g)	38.8 ± 0.1
ABTS scavenging ability (mg TE/g)	36.8 ± 0.4
CUPRAC (mg TE/g)	71.1 ± 1.1
FRAP (mg TE/g)	44.9 ± 2.6
Metal chelating (mg EDTAE/g)	8.9 ± 0.1
Phosphomolybdenum (mmol TE/g)	1.33 ± 0.02
***Enzyme inhibitory properties***	
AChE inhibition (mg GALAE/g)	2.23 ± 0.02
BChE inhibition (mg GALAE/g)	2.38 ± 0.04
Tyrosinase inhibition (mg KAE/g)	60.7 ± 0.6
Amylase inhibition (mmol ACAE/g)	0.41 ± 0.01
Glucosidase inhibition (mmol ACAE/g)	1.62 ± 0.01

Values are reported as mean ± SD of three parallel measurements. GAE: gallic acid equivalent; RE: rutin equivalent; TE: Trolox equivalent; EDTAE: EDTA equivalent; GALAE: galanthamine equivalent; KAE: kojic acid equivalent; ACAE: acarbose equivalent.

## Data Availability

The original contributions presented in the study are included in the article; further inquiries can be directed to the corresponding author.

## Supplementary Materials

The following supporting information can be downloaded at: https://www.mdpi.com/article/10.3390/plants15040522/s1, Table S1: LC-HRMS metabolite profiling of Leucas nubica extract; Figure S1: ESI-MS/MS spectrum of protocatechuic acid (11) at *m*/*z* 153.0193; Figure S2: ESI-MS/MS spectrum of chlorogenic acid (13) at *m*/*z* 353.0878; Figure S3: ESI-MS/MS spectrum of p-coumaric acid (17) at *m*/*z* 163.0401; Figure S4: ESI-MS/MS spectrum of caffeic acid (20) at *m*/*z* 179.0350; Figure S5: ESI-MS/MS spectrum of p-hydroxyphenyl acetic acid (21) at *m*/*z* 151.0400; Figure S6: ESI-MS/MS spectrum of gentisic acid (22) at *m*/*z* 151.0400; Figure S7: ESI-MS/MS spectrum of o-coumaric acid (23) at *m*/*z* 163.0401; Figure S8: ESI-MS/MS spectrum of carboxyverbascoside (32) at *m*/*z* 667.1880; Figure S9: ESI-MS/MS spectrum of echinacoside (31) at *m*/*z* 785.2510; Figure S10: ESI-MS/MS spectrum of leucoseptoside A (41) at *m*/*z* 637.2138; Figure S11: ESI-MS/MS spectrum of alyssonoside (40) at *m*/*z* 769.2561; Figure S12: ESI-MS/MS spectrum of leontoside B (40) at *m*/*z* 783.2717; Figure S13: ESI-MS/MS spectrum of acetylvarbascoside (44) at *m*/*z* 665.2087; Figure S14: ESI-MS/MS spectrum of acetylmartynoside (46) at *m*/*z* 693.2400; Figure S15: ESI-MS/MS spectrum of darendoside B (29) at *m*/*z* 475.1821; Figure S16: ESI-MS/MS spectrum of forsythoside B/samioside/lavandulifolioside (34) at *m*/*z* 755.2404; Figure S17: ESI-MS/MS spectrum of calceolarioside (37) at *m*/*z* 477.1402; Figure S18: ESI-MS/MS spectrum of jionoside C (47) at *m*/*z* 591.2083; Figure S19: Extracted ion chromatogram (EIC) of iridoid and lignan glycosides; Figure S20: ESI-MS/MS spectrum of monotropein (50) at *m*/*z* 389.1089; Figure S21: ESI-MS/MS spectrum of isovitexin (57) at *m*/*z* 431.0984; Figure S22: ESI-MS/MS spectrum of luteolin 7-O-glucoside (61) at *m*/*z* 447.0933; Figure S23: ESI-MS/MS spectrum of apigenin 7-O-glucoside (63) at *m*/*z* 431.0984; Figure S24: ESI-MS/MS spectrum of nepetin (64) at *m*/*z* 315.0510; Figure S25: ESI-MS/MS spectrum of eriodictyol (66) at *m*/*z* 287.0561; Figure S26: ESI-MS/MS spectrum of luteolin (67) at *m*/*z* 285.0405; Figure S27: ESI-MS/MS spectrum of apigenin (72) at *m*/*z* 269.0455; Figure S28: ESI-MS/MS spectrum of cirsiliol (74) at *m*/*z* 329.0667; Figure S29: ESI-MS/MS spectrum of apigenin 7-O-dicoumaroyl-O-hexoside (77) at *m*/*z* 723.1719; Figure S30: ESI-MS/MS spectrum of naringenin 7-O-coumaroyl-O-hexoside (71) at *m*/*z* 579.1508 (mass accuracy 5 ppm) (for numbers and fragmentation patterns, see Table 1) [53].
