# Supplementary material for: LC-MS Profiling and Biological Activity of Unexplored Leucas nubica Benth. (Lamiaceae)
Source: Plants (Basel). 2026 Feb 7;15(4):522. doi: 10.3390/plants15040522 (PMC12944397; doi:10.3390/plants15040522)
Supplement: Supplementary file 1 [file plants-15-00522-s001.zip › plants-4078684-supplementary.pdf]

LC-MS profiling and biological activity of unexplored *Leucas nubica* Benth. (Lamiaceae)

Dimitrina Zheleva-Dimitrova [1*], Gokhan Zengin [2], Sakina Yagi [3], Solafa Suliman [3], and Reneta Gevrenova [1]

[1]Department of Pharmacognosy, Faculty of Pharmacy, Medical University, 1000 Sofia, Bulgaria; dzheleva@pharmfac.mu-sofia.bg (D.Z); rgevrenova@pharmfac.mu-sofia.bg (R.G.);
[2]Physiology and Biochemistry Research Laboratory, Department of Biology, Science Faculty, Selcuk University, Konya 42130, Turkey; gokhanzengin@selcuk.edu.tr (G.Z.);
[3]Department of Botany, Faculty of Science, University of Khartoum, Khartoum, Sudan; sakinayagi@gmail.com (S.Y.); solafa.suliman@gmail.com (S.S.);

***Correspondence: dzheleva@pharmfac.mu-sofia.bg (D.Z.)**

Supplemental material

Total phenolic and flavonoid content

The total phenolic content was determined by employing the methods given in the literature with some modification. Sample solution (0.25 mL) was mixed with diluted Folin–Ciocalteu reagent (1 mL, 1:9, v/v) and shaken vigorously. After 3 min, Na2CO3 solution (0.75 mL, 1%) was added and the sample absorbance was read at 760 nm after a 2 h incubation at room temperature. The total phenolic content was expressed as milligrams of gallic acid equivalents (mg GAE/g extract)[1].

The total flavonoid content was determined using the AlCl3 method. Briefly, sample solution (1 mL) was mixed with the same volume of aluminum trichloride (2%) in methanol. Similarly, a blank was prepared by adding sample solution (1 mL) to methanol (1 mL) without AlCl3. The sample and blank absorbances were read at 415 nm after a 10 min incubation at room temperature. The absorbance of the blank was subtracted from that of the sample. Rutin was used as a reference standard and the total flavonoid content was expressed as milligrams of rutin equivalents (mg RE/g extract) [1]

Determination of Antioxidant and Enzyme Inhibitory Effects

Antioxidant (DPPH and ABTS radical scavenging, reducing power (CUPRAC and FRAP), phosphomolybdenum and metal chelating (ferrozine method)) and enzyme inhibitory activities (cholinesterase (Elmann's method), tyrosinase (dopachrome method), α-amylase (iodine/potassium iodide method), α -glucosidase (chromogenic PNPG method) and pancreatic lipase (*p*-nitrophenyl butyrate (p-NPB) method) were determined using the methods previously described by Uysal et al. [1] and Grochowski et al. [2]

For the DPPH (1,1-diphenyl-2-picrylhydrazyl) radical scavenging assay: Sample solution was added to 4 mL of a 0.004% methanol solution of DPPH. The sample absorbance was read at 517 nm after a 30 min incubation at room temperature in the dark. DPPH radical scavenging activity was expressed as milligrams of trolox equivalents (mg TE/g extract).

For ABTS (2,2′-azino-bis(3-ethylbenzothiazoline) 6-sulfonic acid) radical scavenging assay: Briefly, ABTS+ was produced directly by reacting 7 mM ABTS solution with 2.45 mM potassium persulfate and allowing the mixture to stand for 12–16 in the dark at room temperature. Prior to beginning the assay, ABTS solution was diluted with methanol to an absorbance of 0.700 ± 0.02 at 734 nm. Sample solution was added to ABTS solution (2 mL) and mixed. The sample absorbance was read at 734 nm after a 30 min incubation at room temperature. The ABTS radical scavenging activity was expressed as milligrams of Trolox equivalents (mg TE/g extract).

For CUPRAC (cupric ion reducing activity) activity assay: Sample solution was added to premixed reaction mixture containing CuCl2 (1 mL, 10 mM), neocuproine (1 mL, 7.5 mM) and NH4Ac buffer (1 mL, 1 M, pH 7.0). Similarly, a blank was prepared by adding sample solution (0.5 mL) to premixed reaction mixture (3 mL) without CuCl2 . Then, the sample and blank absorbances were read at 450 nm after a 30 min incubation at room temperature. The absorbance of the blank was subtracted from that of the sample. CUPRAC activity was expressed as milligrams of trolox equivalents (mg TE/g extract).

For FRAP (ferric reducing antioxidant power) activity assay: Sample solution was added to premixed FRAP reagent (2 mL) containing acetate buffer (0.3 M, pH 3.6), 2,4,6- tris(2-pyridyl)-S-triazine (TPTZ) (10 mM) in 40 mM HCl and ferric chloride (20 mM) in a ratio of 10:1:1 (v/v/v). Then, the sample absorbance was read at 593 nm after a 30 min incubation at room temperature. FRAP activity was expressed as milligrams of Trolox equivalents (mg TE/g extract).

For phosphomolybdenum method: Sample solution was combined with 3 mL of reagent solution (0.6 M sulfuric acid, 28 mM sodium phosphate and 4 mM ammonium molybdate). The sample absorbance was read at 695 nm after a 90 min incubation at 95 °C. The total antioxidant capacity was expressed as millimoles of trolox equivalents (mmol TE/g extract).

For metal chelating activity assay: Briefly, sample solution was added to FeCl2 solution (0.05 mL, 2 mM). The reaction was initiated by the addition of 5 mM ferrozine (0.2 mL). Similarly, a blank was prepared by adding sample solution (2 mL) to FeCl2 solution (0.05 mL, 2 mM) and water (0.2 mL) without ferrozine. Then, the sample and blank absorbances were read at 562 nm after 10 min incubation at room temperature. The absorbance of the blank was subtracted from that of the sample. The metal chelating activity was expressed as milligrams of EDTA (disodium edetate) equivalents (mg EDTAE/g extract).

For Cholinesterase (ChE) inhibitory activity assay: Sample solution (was mixed with DTNB (5,5-dithio-bis(2-nitrobenzoic) acid, Sigma, St. Louis, MO, USA) (125 μL) and AChE (acetylcholines-terase (Electric ell acetylcholinesterase, Type-VI-S, EC 3.1.1.7,Sigma)), or BChE (butyrylcholinesterase (horse serum butyrylcholinesterase, EC 3.1.1.8, Sigma)) solution (25 μL) in Tris–HCl buffer (pH 8.0) in a 96-well microplate and incubated for 15 min at 25 °C. The reaction was then initiated with the addition of acetylthiocholine iodide (ATCI, Sigma) or butyrylthiocholine chloride (BTCl, Sigma) (25 μL). Similarly, a blank was prepared by adding sample solution to all reaction reagents without enzyme (AChE or BChE) solution. The sample and blank absorbances were read at 405 nm after 10 min incubation at 25 °C. The absorbance of the blank was subtracted from that of the sample and the cholinesterase inhibitory activity was expressed as galanthamine equivalents (mgGALAE/g extract).

For Tyrosinase inhibitory activity assay: Sample solution was mixed with tyrosinase solution (40 μL, Sigma) and phosphate buffer (100 μL, pH 6.8) in a 96-well microplate and incubated for 15 min at 25 °C. The reaction was then initiated with the addition of L-DOPA (40 μL, Sigma). Similarly, a blank was prepared by adding sample solution to all reaction reagents without enzyme (tyrosinase) solution. The sample and blank absorbances were read at 492 nm after a 10 min incubation at 25 °C. The absorbance of the blank was subtracted from that of the sample and the tyrosinase inhibitory activity was expressed as kojic acid equivalents (mgKAE/g extract).

For α-amylase inhibitory activity assay: Sample solution was mixed with α- amylase solution (ex-porcine pancreas, EC 3.2.1.1, Sigma) (50 μL) in phosphate buffer (pH 6.9 with 6 mM sodium chloride) in a 96-well microplate and incubated for 10 min at 37 °C. After pre-incubation, the reaction was initiated with the addition of starch solution (50 μL, 0.05%). Similarly, a blank was prepared by adding sample solution to all reaction reagents without enzyme (α-amylase) solution. The reaction mixture was incubated 10 min at 37 °C. The reaction was then stopped with the addition of HCl (25 μL, 1 M). This was followed by addition of the iodine-potassium iodide solution (100 μL). The sample and blank absorbances were read at 630 nm. The absorbance of the blank was subtracted from that of the sample and the α-amylase inhibitory activity was expressed as acarbose equivalents (mmol ACE/g extract).

For α-glucosidase inhibitory activity assay: Sample solution was mixed with glutathione (50 μL), α-glucosidase solution (from Saccharomyces cerevisiae, EC 3.2.1.20, Sigma) (50 μL) in phosphate buffer (pH 6.8) and PNPG (4-N-trophenyl-α-Dglucopyranoside,
Sigma) (50 μL) in a 96-well microplate and incubated for 15 min at 37 °C. Similarly, a blank was prepared by adding sample solution to all reaction reagents without enzyme (α-glucosidase) solution. The reaction was then stopped with the addition of sodium carbonate (50 μL, 0.2 M). The sample and blank absorbances were read at 400 nm. The absorbance of the blank was subtracted from that of the sample and the α-glucosidase inhibitory activity was expressed as acarbose equivalents (mmol ACE/g extract).

Table S1. LC-HRMS metabolite profiling of *Leucas nubica* extract

№	Identified/Tentatively Annotated Compound	Molecular Formula	Exact Mass [M-H]⁻	Fragmentation pattern in (-) ESI-MS/MS	tR (min)	Δ ppm	Level of confidence [16]
				**Sugar acids and saccharides**			
**1.**	xylonic acid	$C_5H_{10}O_6$	165.0405	165.0394 (42.0), 147.0287 (8.4), 129.0181 (8.3), 111.0073 (0.5), 105.0179 (10.1), 101.00299 (2.5), 87.0072 (17.6), 75.0072 (5.6)	0.73	−6.188	D1
**2.**	hexose	$C_6H_{12}O_6$	179.0561	179.0552 (48.63), 161.0444 (11.6), 143.0337 (9.9), 125.0229 (1.6), 99.0436 (2.0), 81.0330 (6.1), 75.0071 (100)	0.79	−5.201	D1
**3.**	gluconic acid	$C_6H_{12}O_7$	195.0510	195.0503 (25.8), 177.0394 (4.9), 159.0289 (3.4), 141.0181 (1.0), 129.0180 (41.4), 105.0177 (2.3), 75.0071 (100)	0.74	-3.824	D1
**4.**	asystoside/ebracteatoside B/lunaroside	$C_{25}H_{44}O_{15}$	583.2607	583.2601 (100), 421.2077 (6.5), 289.1657 (14.8), 161.0445 (16.6), 101.0229 (25.4), 71.0122 (40.4)	7.12	-1.155	D2
				**Carboxylic acids**			
**5.**	malic acid[a]	$C_4H_6O_5$	133.0142	133.0131 (14.7), 115.0023 (97.1), 89.0229 (10.2), 72.9915 (27.2), 71.0122 (100)	0.81	-8.921	B
**6.**	citric /isocitric acid	$C_6H_8O_7$	191.0197	191.0190 (6.9), 173.0446 (1.1), 154.9975 (0.7), 147.0287 (0.7), 129.0180 (5.1), 111.0073 (100), 101.0231 (0.3), 87.0072 (48.1), 85.0280 (28.1)	1.11	-4.061	D1
**7.**	oxaloglutaric acid	$C_7H_8O_7$	203.0197	203.0190 (73.2), 159.0285 (1.0), 97.0280 (100), 95.0124 (14.1), 141.0182 (29.3), 115.0022 (11.0)	1.13	-3.526	D1
**8.**	quinic acid	$C_7H_{12}O_6$	191.0561	191.0552 (50.3), 145.0494 (13.9), 129.0544 (62.3), 115.0386 (82.7), 101.0593 (100), 87.0435 (41.5), 85.0643 (17.0)	2.34	-4.665	D1
				**Hydroxybenzoic, hydroxycinnamic, acylquinic acids, and their derivatives**			
**9.**	salvianic acid A	$C_9H_{10}O_5$	197.0455	197.0447 (8.2), 179.0340 (44.5), 163.0233 (0.3), 151.0391 (2.9), 135.0438 (71.9), 123.0438 (59.0), 117.0333 (0.6), 109.0281 (4.1), 72.9915 (100)	2.63	-4.246	D1
**10.**	dihydrocaffeic acid O-hexoside	$C_{15}H_{20}O_9$	343.1035	343.1034 (97.8), 223.0612 (5.4), 181.0497 (80.5), 163.0.90 (19.3), 121.0278 (2.3), 119.0337 (20.6), 109.0643 (1.1), 59.0123 (24.5)	2.71	-0.278	D2
**11.**	protocatechuic acid[a]	$C_7H_6O_4$	153.0193	153.0181 (13.5), 109.0280 (100), 123.0436 (1.1)	2.95	-7.790	B
**12.**	vanillyl alcohol	$C_8H_{10}O_3$	153.0557	153.0545 (13.8), 137.0233 (0.8), 123.0438 (100), 109.0281 (4.1), 95.0487 (2.1),	3.07	-7.594	D1
**13.**	chlorogenic acid[a]	$C_{16}H_{18}O_9$	353.0878	353.0885 (6.5), 191.0189 (100), 179.0341 (12.0), 161.0233 (2.9), 135.0438 (9.9), 85.0279 (58.0),	3.19	1.967	B
**14.**	peiioside B	$C_{25}H_{38}O_{16}$	593.2087	593.2084 (100), 461.1665 (14.9), 315.1086 (5.7), 297.0976 (1.8), 279.0890 (0.2), 191.0553 (1.6), 179.0557 (0.2), 161.0447 (2.8), 135.0439 (26.1), 113.0230 (30.6)	3.26	-0.570	D2
**15.**	caffeic acid *O*-rutinoside (swertiamacroside)	$C_{21}H_{28}O_{13}$	487.1457	487.1457 (19.8), 179.0340 (100), 161.0234 (14.5), 135.0439 (44.6), 85.0280 (3.1)	3.50	-0.049	D1
**16.**	dihydroxybenzoic acid	$C_7H_6O_4$	153.0193	153.0182 (37.2), 109.0285 (18.3), 108.0202 (100), 95.0123 (30.3), 85.0289 (27.4)	4.14	-7.594	D2
**17.**	*p*-coumaric acid[a]	$C_9H_8O_3$	163.0401	163.0390 (5.2), 119.0488 (100), 93.0331 (1.2)	4.19	-6.792	B

**18.**	*p*-coumaric acid *O*-hexoside	$C_{15}H_{18}O_8$	325.0928	325.0909 (1.0), 163.0390 (100), 119.0489 (87.3), 145.0283 (2.9)	4.20	-6.277	D1
**19.**	ferulic acid *O*-hexosyl-deoxyhexoside	$C_{22}H_{30}O_{13}$	501.1614	501.1610 (6.7), 339.1062 (0.3), 357.1180 (2.1), 193.0498 (100), 175.0391 (7.9), 160.0155 (10.1), 134.0360 (39.8), 178.0259 (1.1), 149.0596 (4.5), 113.0231 (6.9)	4.57	-0.647	D1
**20.**	*p*-hydroxyphenyl acetic acid[a]	$C_8H_8O_3$	151.0400	151.0389 (100), 136.0151 (0.6), 123.0074 (4.1), 109.0280 (16.3)	4.59	-7.464	B
**21.**	caffeic acid[a]	$C_9H_8O_4$	179.0350	179.0341 (15.9), 135.0439 (100), 117.0331 (0.8), 107.0489 (1.3)89.0382 (0.5)	4.61	-5.094	B
**22.**	gentisic acid[a]	$C_7H_6O_4$	153.0193	153.0182 (35.9), 123.0439 (0.3), 109.0281 (100), 108.0202 (8.8)	4.91	−7.594	B
**23.**	*o*-coumaric acid[a]	$C_9H_8O_3$	163.0401	163.0389 (5.7), 119.0488 (100), 93.0332 (1.3)	5.72	-6.915	B
**24.**	dicaffeoylhexose	$C_{24}H_{24}O_{12}$	503.1195	503.1195 (68.9), 341.0894 (2.3), 323.0776 (4.7), 203.0341 (8.1), 179.0340 (98.9), 161.0233 (21.1), 135.0438 (100)	6.38	0.081	D2
**25.**	syringalide A-deoxyhexoside	$C_{29}H_{36}O_{14}$	607.2032	607.2030 (67.0), 445.1702 (1.0), 299.1149 (0.5), 137.0595 (0.2), 179.0341 (0.2), 161.0233 (100), 135.0438 (4.2), 133.0282 (34.1)	6.83	-0.377	D2
**26.**	dicaffeoylhexose	$C_{24}H_{24}O_{12}$	503.1195	503.1196 (94.5), 341.0875 (3.1), 323.0772 (21.2), 281.0658 (2.4), 251.0555 (2.0), 221.0453 (5.2), 203.0346 (1.2), 179.03410 (82.5), 161.0223 (53.1), 135.0439 (100)	7.03	0.141	D2
**27.**	hydroxybenzoic acid	$C_7H_6O_3$	137.0244	137.0232 (8.1), 93.0330 (100)	7.98	-9.030	D2
	**Phenylethanoid glycosides**						
**28.**	decaffeoyl aceteoside/ decaffeoyl verbasoside	$C_{20}H_{30}O_{12}$	461.1664	461.1663 (100), 315.1087 (4.7), 297.0984 (1.6), 161.0447 (2.9), 153.0546 (1.7), 135.0439 (28.4), 123.0438 (2.4), 113.0230 (43.0), 95.0123 (7.5), 85.0279 (20.6)	3.28	-0.346	D1
**29.**	darendoside B	$C_{21}H_{32}O_{12}$	475.1821	475.1820 (100), 329.1244 (3.3), 311.1146 (0.2), 149.0596 (9.3), 179.0553 (0.7), 167.0704 (0.9), 161.0445 (6.7), 134.0361 (7.5), 113.0230 (54.4)	4.26	-0.252	D2
**30.**	hydroxyverbascoside	$C_{29}H_{36}O_{16}$	639.1931	639.1925 (78.2), 621.1810 (5.9), 459.1501 (1.4), 179.0340 (40.3), 161.0233 (100), 151.0389 (17.4), 135.0438 (24.1), 1330282 (36.1), 113.0230 (11.1)	5.17	-0.873	D1
**31.**	echinacoside	$C_{35}H_{46}O_{20}$	785.2510	785.2502 (54.8), 623.1982 (19.6), 461.1663 (10.6), 179.0341 (2.7), 161.0234 (100), 135.0439 (14.7), 133.0282 (37.5), 113.0230 (7.1)	5.60	-0.926	D1
**32.**	carboxyverbascoside	$C_{30}H_{36}O_{17}$	667.1880	667.1879 (83.8), 623.1962 (5.9), 487.1456 (0.9), 461.1667 (8.4), 315.1082 (1.5), 179.0340 (45.2), 161.0233 (100), 153.0539 (0.7), 135.0439 (53.8), 133.0282 (33.4), 113.0231 (13.1), 85.0280 (6.5)	5.65	-0.064	E
**33.**	hydroxyverbascoside isomer	$C_{29}H_{36}O_{16}$	639.1931	639.1920 (85.4), 621.1811 (4.7), 179.0341 (37.9), 135.0439 (22.0), 161.0233 (100), 151.0390 (17.1), 135.0439 (22.0), 113.0228 (11.5)	5.86	-1.624	D1
**34.**	forsythoside B/samioside/lavandulifolioside	$C_{34}H_{44}O_{19}$	755.2404	755.2404 (81.2), 593.2090 (6.7), 461.1664 (7.7), 297.0986 (0.5), 179.0340 (5.4), 161.0234 (100), 135.0438 (21.8), 133.0282 (49.8), 113.0230 (9.5)	6.05	-0.056	D1
**35.**	echinacoside isomer	$C_{35}H_{46}O_{20}$	785.2510	785.2505 (87.1), 623.2186 (8.4), 461.1690 (2.8), 179.0341 (2.9), 161.0233 (100), 135.0437 (13.9), 133.0281 (45.7), 113.0231 (16.9)	6.08	-0.531	D1
**36.**	verbascoside[a]	$C_{29}H_{36}O_{15}$	623.1981	623.1978 (60.5), 461.1664 (6.9), 315.1084 (1.8), 179.0342 (2.6), 161.0234 (100), 153.0544 (0.4), 135.0439 (11.4), 133.0282 (32.9), 113.0230 (9.2)	6.24	-0.631	C

**37.**	calceolarioside	$C_{23}H_{26}O_{11}$	477.1402	477.1398 (100), 323.0771 (23.6), 315.1089 (0.9), 179.0341 (11.5), 161.0234 (91.4), 153.0546 (42.3), 135.0439 (15.4), 133.0282 (36.2), 123.0438 (44.9)	6.43	-0.911	D1
**38.**	forsythoside B/samioside/lavandulifolioside	$C_{34}H_{44}O_{19}$	755.2404	755.2401 (93.2), 593.2095 (10.5), 461.1666 (9.1), 297.0967 (0.4), 179.0341 (7.6), 161.0234 (100), 135.0439 (27.2), 133.0282 (47.3), 113.0230 (13.8)	6.48	-0.387	D1
**39.**	isoverbascoside	$C_{29}H_{36}O_{15}$	623.1981	623.1976 (100), 461.1663 (10.9), 315.1090 (2.2), 179.0338 (4.0), 161.0233 (96.2), 135.0439 (16.8), 133.0282 (35.9), 113.0229 (11.8)	6.61	-0.920	D1
**40.**	alyssonoside	$C_{35}H_{46}O_{19}$	769.2561	769.2554 (100), 637.2224 (0.7), 593.2086 (5.7), 461.1667 (7.8), 315.1098 (2.2), 297.0981 (0.8), 193.0499 (1.0), 175.0392 (47.3), 161.0444 (1.7), 160.0155 (47.5), 161.0235 (15.4), 153.0546 (6.0), 149.0597 (1.9), 132.024 (15.3), 113.0230 (14.5)	6.83	-0.809	D1
**41.**	leucoseptoside A	$C_{30}H_{38}O_{15}$	637.2138	637.2136 (100), 461.1662 (12.3), 315.1092 (3.9), 193.0500 (13.0), 175.0392 (79.1), 60.0155 (48.5), 161.0235 (28.6), 161.0443 (1.5), 153.0548 (7.4), 149.0596 (15.3), 132.0204 (16.9), 113.0230 (20.6),	7.07	-0.272	D1
**42.**	leontoside B/stachyoside D	$C_{36}H_{48}O_{19}$	783.2717	783.2708 (100), 607.2193 (1.2), 505.1721 (0.5), 475.1816 (1.3), 461.1661 (0.4), 329.1273 (0.4), 193.0499 (34.8), 175.0391 (84.3), 167.0707 (0.6), 161.0448 (2.1), 161.0225 (5.6), 160.0155 (63.4), 135.0434 (1.3), 134.0361 (35.6), 113.0230 (16.3)	7.40	-1.139	D1
**43.**	leucoseptoside A isomer	$C_{30}H_{38}O_{15}$	637.2138	637.2135 (100), 461.1665 (11.7), 315.1100 (2.5), 193.0497 (11.1), 175.0390 (47.9), 160.0154 (33.5), 161.0232 (31.9), 161.0443 (1.5), 153.0547 (0.9), 149.0593 (0.9), 132.0204 (8.4), 113.0230 (16.9)	7.58	-0.461	D1
**44.**	acetylverbascoside	$C_{31}H_{38}O_{16}$	665.2087	665.2085 (72.6), 503.1794 (2.9), 461.1692 (2.7), 179.0341 (1.2), 161.0253 (100), 135.0439 (11.6), 133.0282 (34.9), 113.0228 (4.1)	7.84	-0.313	D2
**45.**	martynoside	$C_{31}H_{40}O_{15}$	651.2294	651.2294 (94.8), 475.1845 (0.8), 329.1237 (0.3), 193.0499 (13.6), 175.0391 (100), 161.0232 (6.9), 160.0155 (62.7), 132.0203 (24.0), 113.0230 (12.3)	7.88	-0.113	D1
**46.**	acetylmartynoside	$C_{33}H_{42}O_{16}$	693.2400	693.2393 (93.4), 651.2286 (0.9), 475.1799 (0.6), 329.1257 (0.6), 193.0497 (15.7), 175.0391 (100), 161.0199 (2.7), 160.0155 (67.9), 132.0204 (27.1), 113.0229 (7.3)	8.68	-0.993	D2
**47.**	jionoside C	$C_{29}H_{36}O_{13}$	591.2083	591.2079 (67.1), 161.0234 (100), 179.0342 (1.9), 135.0439 (4.9), 133.0282 (34.3), 113.0230 (10.9)	8.80	0.210	D2
**48.**	acetylmartynoside isomer	$C_{33}H_{42}O_{16}$	693.2400	693.2398 (92.2), 651.2318 (1.6), 475.1815 (0.8), 193.0499 (14.8), 175.0391 (100), 161.0189 (3.3), 160.0155 (69.1), 132.0204 (26.2), 113.0229 (6.6)	9.01	-0.286	D2
				**Iridoid and lignan glycosides**			
**49.**	geniposidic acid	$C_{16}H_{22}O_{10}$	373.1140	373.1138 (32.0), 211.0606 (50.3), 193.0502 (3.6), 167.0703 (25.8), 149.0596 (54.4), 123.0438 (100), 105.0331 (6.9)	2.70	-0.670	D2
**50.**	monotropein	$C_{16}H_{22}O_{11}$	389.1089	389.1089 (100), 345.1190 (56.2), 227.0538 (5.4), 209.0452 (15.8), 191.0186 (6.4), 183.0653 (48.9), 165.0546 (41.7), 147.0290 (2.5), 139.0387 (60.4), 113.0231 (6.8), 121.0644 (23.1), 101.0230 (5.2), 65.0380 (38.9)	3.06	2.27	D2
**51.**	secoisolariciresinol O-hexoside	$C_{26}H_{36}O_{11}$	523.2185	523.2182 (9.3), 361.1657 (100), 346.1420 (5.1), 313.1462 (0.6), 223.0974 (0.2), 179.0703 (3.1), 165.0547 (7.9)	5.71	-0.545	D1

**52.**	caffeoyl-mussaenosidic acid	$C_{25}H_{30}O_{13}$	537.1614	537.1611 (100), 493.1713 (0.9), 375.1089 (1.0), 357.1001 (0.9), 331.1187 (16.4), 313.1083 (11.4), 213.0762 (1.1), 287.0929 (0.8), 243.1017 (0.6), 213.0762 (1.1), 195.0649 (1.1), 179.0340 (64.3), 135.0438 (70.6), 161.0233 (0.6), 169.0886 (0.2), 151.0751 (1.7), 107.0487 (4.9)	6.90	-0.547	D2
**53.**	syringaresinol O-hexoside	$C_{28}H_{36}O_{13}$	579.2083	579.2076 (3.4), 417.1553 (100), 402.1319 (10.6), 387.1092 (4.3), 359.1153 (0.6), 181.0497 (64.4), 166.0260 (33.5), 151.0024 (17.9), 123.0073 (1.8), 109.0278 (1.8)	6.91	-1.164	D1
				**Flavonoids**			
**54.**	naringenin 6,8-*C*-dihexoside	$C_{27}H_{32}O_{15}$	595.1668	595.1666 (100), 475.1238 (3.7), 457.1166 (0.9), 415.1032 (10.8), 385.0928 (33.2), 355.0820 (35.1), 271.0619 (0.7), 163.0031 (0.6), 151.0025 (2.0), 119.0488 (15.9), 107.0125 (3.5)	4.36	-0.358	D1
**55.**	apigenin 6,8-*C*-hexosyl hexoside	$C_{27}H_{30}O_{15}$	593.1512	593.1508 (100), 503.1196 (4.2), 473.1086 (14.0), 413.0877 (2.6), 395.0765 (2.3), 383.0771 (21.7), 353.0666 (35.9), 325.0715 (2.3), 297.0769 (10.8), 161.0235 (2.0), 117.0333 (3.9)	4.70	-0.730	D1
**56.**	luteolin 7-*O*-dihexoside	$C_{27}H_{30}O_{16}$	609.1461	609.1456 (100), 447.0934 (4.5), 429.0834 (1.0), 285.0403 (61.9), 227.0343 (1.4), 151.0025 (5.3), 133.0282 (5.1), 107.0123 (2.9)	5.62	-0.784	D2
**57.**	isovitexin[a]	$C_{21}H_{20}O_{10}$	431.0984	431.0979 (100), 341.0664 (10.8), 311.0563 (94.9), 283.0612 (28.2), 269.0458 (0.9), 135.0439 (3.5), 117.0331 (13.5), 109.0274 (0.6)	5.99	-1.021	B
**58.**	apigenin-*O*-deoxyhexosylhexoside	$C_{27}H_{28}O_{16}$	607.1305	607.1301 (45.2), 269.0459 (100), 225.0555 (1.4), 197.0603 (0.9), 149.0236 (1.1), 117.0331 (5.5), 151.0026 (1.1), 107.0124 (2.4)	6.11	0.540	D1
**59.**	apigenin *O*-hexosyl-hexoside	$C_{27}H_{30}O_{15}$	593.1512	593.1508 (58.3), 431.0988 (1.6), 413.0866 (0.8), 269.0456 (100), 251.0346 (0.2), 225.0553 (1.6), 211.0395 (0.5), 161.0233 (1.0), 151.0024 (1.8), 117.0333 (4.4), 107.0124 (2.3)	6.24	-0.629	D1
**60.**	luteolin 7-*O*-hexuronide	$C_{27}H_{30}O_{16}$	461.0725	461.0722 (49.9), 285.0405 (100), 243.0285 (0.8), 151.0025 (4.6), 133.0282 (10.6), 107.0122 (2.4)	6.23	0.519	D1
**61.**	luteolin 7-*O*-glucoside[a]	$C_{21}H_{20}O_{11}$	447.0933	447.0930 (100), 285.0402 (91.5), 284.0326 (36.6), 227.0339 (1.6), 211.0390 (1.2), 151.0025 (5.8), 133.0281 (5.5), 151.0025 (5.8), 133.0281 (5.5), 107.0123 (3.8)	6.24	-0.525	B
**62.**	luteolin 4'-*O*-hexoside	$C_{21}H_{20}O_{10}$	447.0933	447.0929 (21.5), 285.0406 (100), 284.0318 (0.4), 151.0027 (6.1), 133.0283 (11.2), 107.0125 (2.6)	6.99	-0.793	D1
**63.**	apigenin 7-*O*-glucoside[a]	$C_{21}H_{20}O_{10}$	431.0984	431.0980 (100), 341.0686 (0.2), 311.0576 (1.4), 268.0374 (63.2), 211.0392 (2.0), 151.0026 (3.7), 107.0123 (2.6), 117.0331 (1.6), 107.0123 (2.6)	7.00	-0.812	B
**64.**	nepetin[a]	$C_{16}H_{12}O_{7}$	315.0510	315.0511 (100), 300.0275 (60.5), 271.0235 (1.1), 271.0235 (1.1), 255.0291 (0.7), 227.0345 (2.3), 201.0184 (4.2), 165.9899 (1.3), 136.9867 (8.8)	7.95	0.299	B
**65.**	cirsiliol-*O*-hexoside	$C_{23}H_{24}O_{12}$	491.1195	491.1200 (27.1), 329.0667 (100), 314.0434 (0.9), 313.0362 (6.8), 299.0198 (27.5), 271.0241 (3.7), 300.0224 (1.7), 243.0288 (3.0), 227.0337 (2.0), 163.0022 (1.9), 133.0283 (4.5), 178.9984 (0.4), 151.0021 (0.7)	8.29	1.020	D1
**66.**	eriodictyol[a]	$C_{15}H_{12}O_{6}$	287.0561	287.0562 (12.5), 151.0025 (100), 135.0438 (92.8), 125.0229 (3.9), 109.0280 (2.1), 107.0123 (12.8)	8.68	0.239	B

**67.**	luteolin[a]	$C_{15}H_{10}O_6$	285.0405	285.0404 (100), 256.0338 (0.2), 241.0503 (0.7), 229.0511 (0.1), 217.0503 (1.0), 151.0025 (4.4), 133.0282 (23.6), 121.0281 (1.2), 107.0124 (4.1)	8.76	-0.075	B
**68.**	apigenin 7-*O*-coumaroylhexoside	$C_{30}H_{26}O_{12}$	577.1351	577.1346 (65.5), 431.0982 (1.0), 307.0827 (0.8), 269.0456 (100), 225.0553 (1.6), 145.0282 (13.7), 163.0384 (1.0), 227.0339 (0.7), 119.0486 (1.4), 117.0331 (11.8), 151.0024 (1.3), 149.0230 (1.1), 107.0122 (2.1)	9.26	-0.883	D1
**69.**	chrysoeriol/diosmetin O-hexoside	$C_{22}H_{22}O_{11}$	461.1089	461.1088 (19.3), 299.0560 (100), 284.0325 (40.2), 256.0375 (2.1), 227.0348 (0.7), 211.0389 (0.3), 151.0023 (0.7), 133.0284 (1.1), 107.0122 (0.3)	9.27	-0.379	D1
**70.**	apigenin 7-*O*-coumaroylhexoside isomer	$C_{30}H_{26}O_{12}$	577.1351	577.1347 (52.3), 431.0985 (0.9), 307.0818 (0.8), 269.0455 (100), 225.0553 (1.7), 227.0353 (0.5), 163.0387 (0.8), 151.0026 (1.4), 149.0231 (1.2), 145.0282 (13.5), 119.0487 (1.9), 117.0331 (12.4), 107.0124 (2.5)	9.57	-0.779	D1
**71.**	naringenin 7-*O*-coumaroylhexoside	$C_{30}H_{28}O_{12}$	579.1508	579.1505 (63.9), 271.0613 (100), 227.0706 (1.3), 163.0389 (2.6), 151.0024 (41.1), 145.0282 (13.1), 119.0488 (31.1), 107.0123 (14.4)	9.80	-0.500	D1
**72.**	apigenin[a]	$C_{15}H_{10}O_5$	269.0455	269.0457 (100), 225.0551 (2.0), 151.0025 (5.5), 149.0231 (4.9), 117.0332 (19.4), 107.0123 (4.9)	9.91	0.384	B
**73.**	naringenin 7-O-coumaroylhexoside isomer	$C_{30}H_{28}O_{12}$	579.1508	579.1506 (58.8), 271.0612 (100), 227.0717 (1.3), 163.0384 (2.2), 151.0025 (38.5), 145.0283 (13.9), 119.0488 (32.7), 107.0123 (14.6)	10.13	-0.396	D1
**74.**	cirsiliol[a]	$C_{17}H_{14}O_7$	329.0667	329.0667 (100), 314.0433 (43.5), 299.0197 (40.6), 285.0405 (4.4), 271.0245 (5.3), 227.0349 (3.7), 199.0394 (14.1), 163.0025 (3.5), 136.9864 (1.4), 133.0282 (15.0)	10.50	0.103	B
**75.**	diosmetin	$C_{16}H_{12}O_6$	299.0561	299.0561 (100), 284.0327 (46.7), 256.0367 (3.8), 227.0346 (1.6), 211.0395 (1.4), 151.0024 (2.9), 133.0282 (4.3), 107.0124 (2.7)	11.70	-0.171	D1
**76.**	velutin	$C_{17}H_{14}O_6$	313.0718	313.0717 (100), 298.0481 (50.8), 283.0246 (54.7), 255.0293 (9.9), 227.0354 (4.5), 211.0383 (1.2), 163.0025 (11.9), 178.9984 (0.7), 151.0024 (0.9), 135.0078 (4.5), 117.0332 (11.9), 136.9864 (1.0)	11.86	-0.036	D1
**77.**	apigenin 7-*O*-dicoumaroyl-*O*-hexoside	$C_{39}H_{32}O_{14}$	723.1719	723.1708 (59.9), 559.1234 (2.9), 269.0455 (100), 225.0556 (1.5), 163.0390 (6.3), 145.0282 (56.9), 117.0332 (38.7), 151.0025 (1.9), 107.0123 (2.5)	12.36	-0.830	D2
**78.**	apigenin 7-*O*-dicoumaroyl-*O*-hexoside isomer	$C_{39}H_{32}O_{14}$	723.1719	723.1715 (62.7), 559.1252 (2.6), 269.0456 (100), 225.0552 (2.0), 163.0390 (6.0), 145.0283 (54.2), 151.0025 (1.7), 149.0234 (2.2), 117.0332 (37.4), 107.0123 (2.4)	12.55	0.179	D2

$[M\text{-}H]^-$ -deprotonated molecular ion; Exact mass: calculated mass of an ion whose elemental formula, isotopic composition and charge state are known, i.e., it is the theoretical mass; t_R: retention time; Δ ppm: delta parts per million-a measurement of the mass accuracy, or the difference between an experimentally measured mass and its theoretically calculated mass; Confidence level: B: confirmed structure except for one or more stereochemical aspects; C: tentative identification matched with a standard compound, match of at least tR, MS and MS/MS with an actual authentic standard analyzed in parallel, preferably supported by other online data; D: Tentative identification based on libraries, model compounds etc.; D1: relatively reliable evidence; D2: relatively poor evidence; E: tentative candidate or tentative identification of metabolite class [16]; [a]-compare to reference standard;

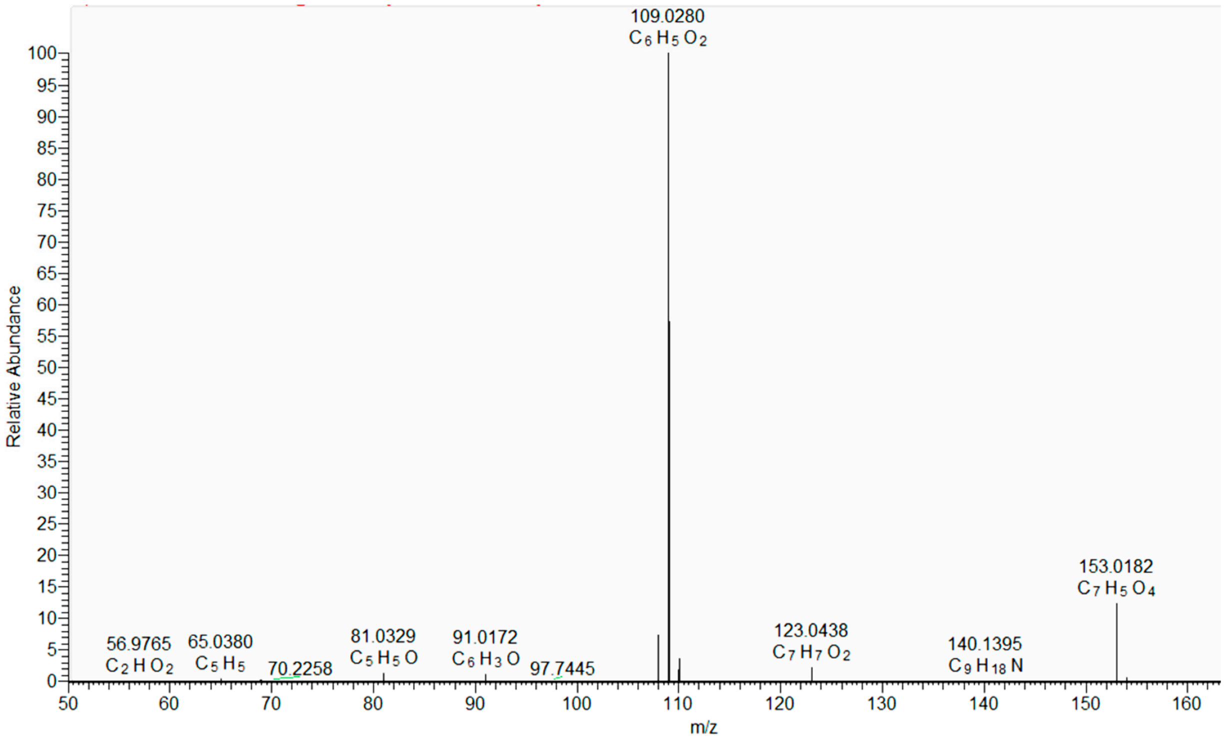

Figure S1. ESI-MS/MS spectrum of protocatechuic acid (11) at *m*/*z* 153.0193 (mass accuracy 5 ppm) (for numbers and fragmentation patterns, see Table 1).

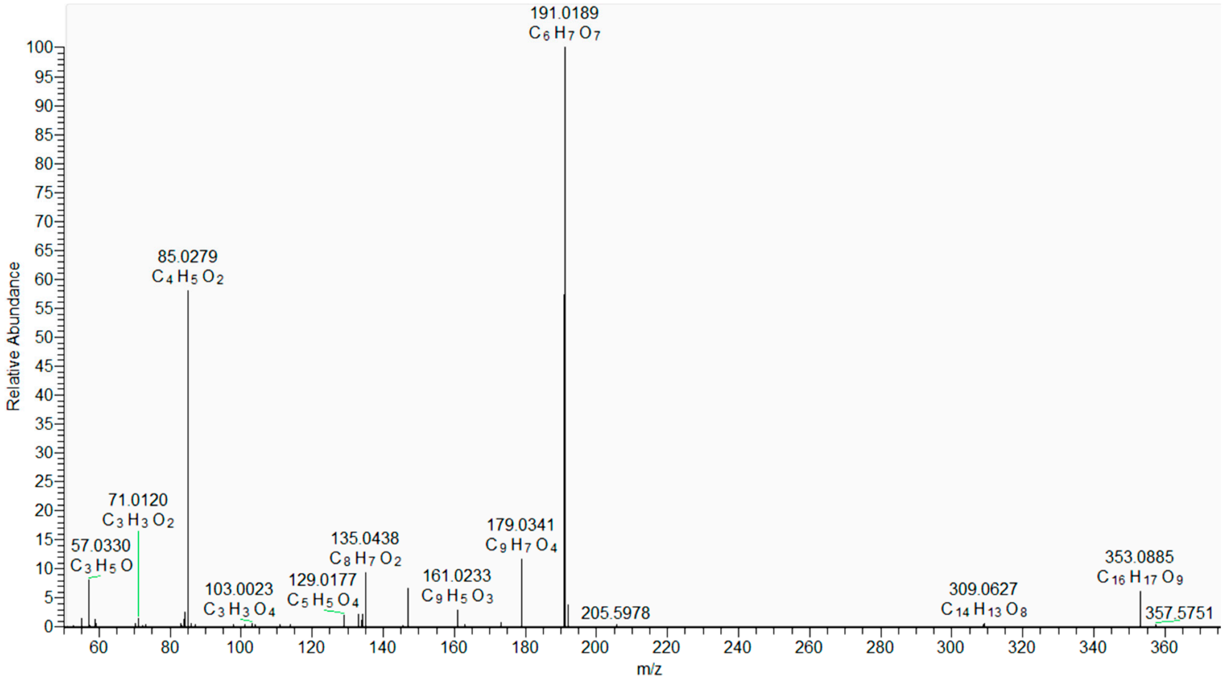

Figure S2. ESI-MS/MS spectrum of chlorogenic acid (13) at *m*/*z* 353.0878 (mass accuracy 5 ppm) (for numbers and fragmentation patterns, see Table 1).

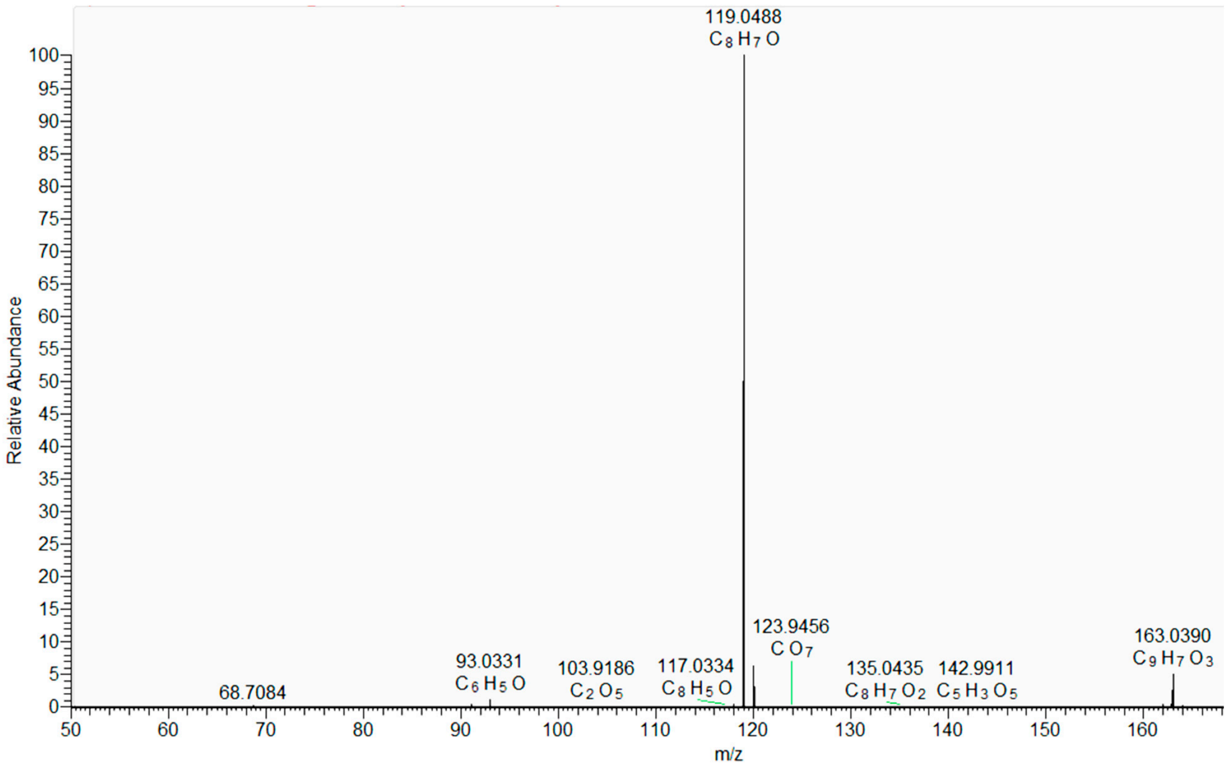

Figure S3. ESI-MS/MS spectrum of *p*-coumaric acid (17) at *m*/*z* 163.0401 (mass accuracy 5 ppm) (for numbers and fragmentation patterns, see Table 1).

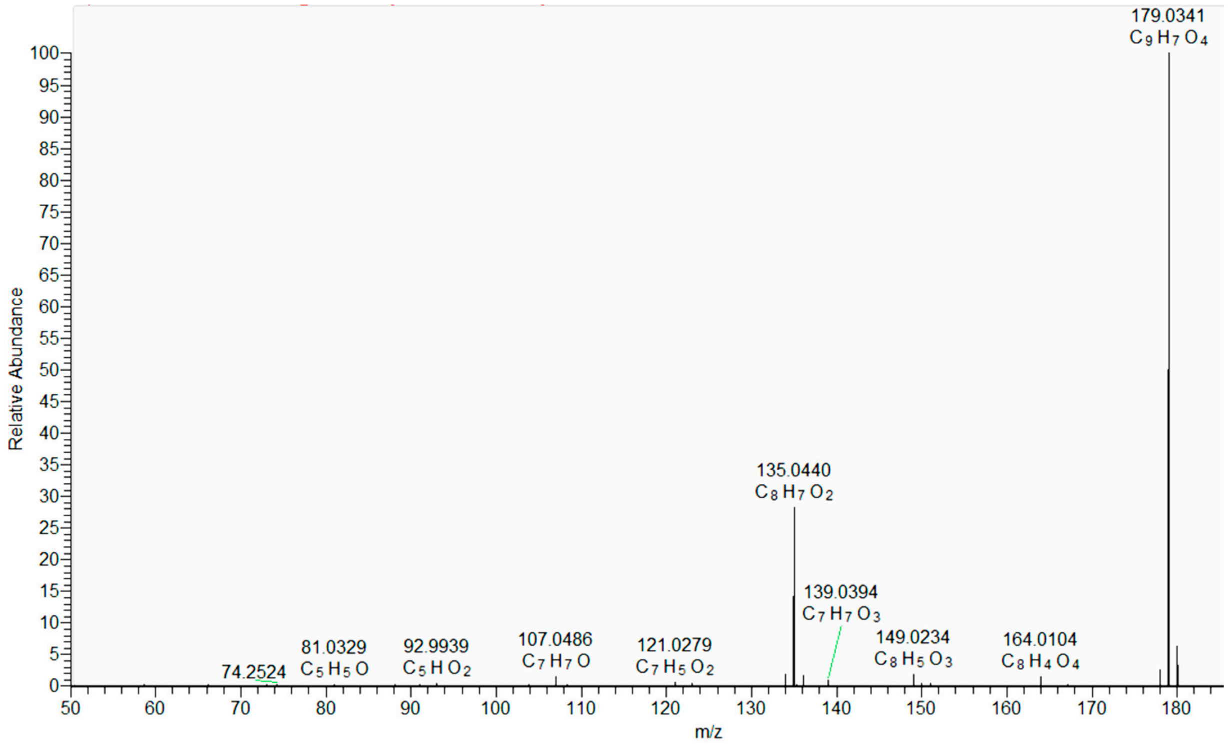

Figure S4. ESI-MS/MS spectrum of caffeic acid (20) at *m*/*z* 179.0350 (mass accuracy 5 ppm) (for numbers and fragmentation patterns, see Table 1).

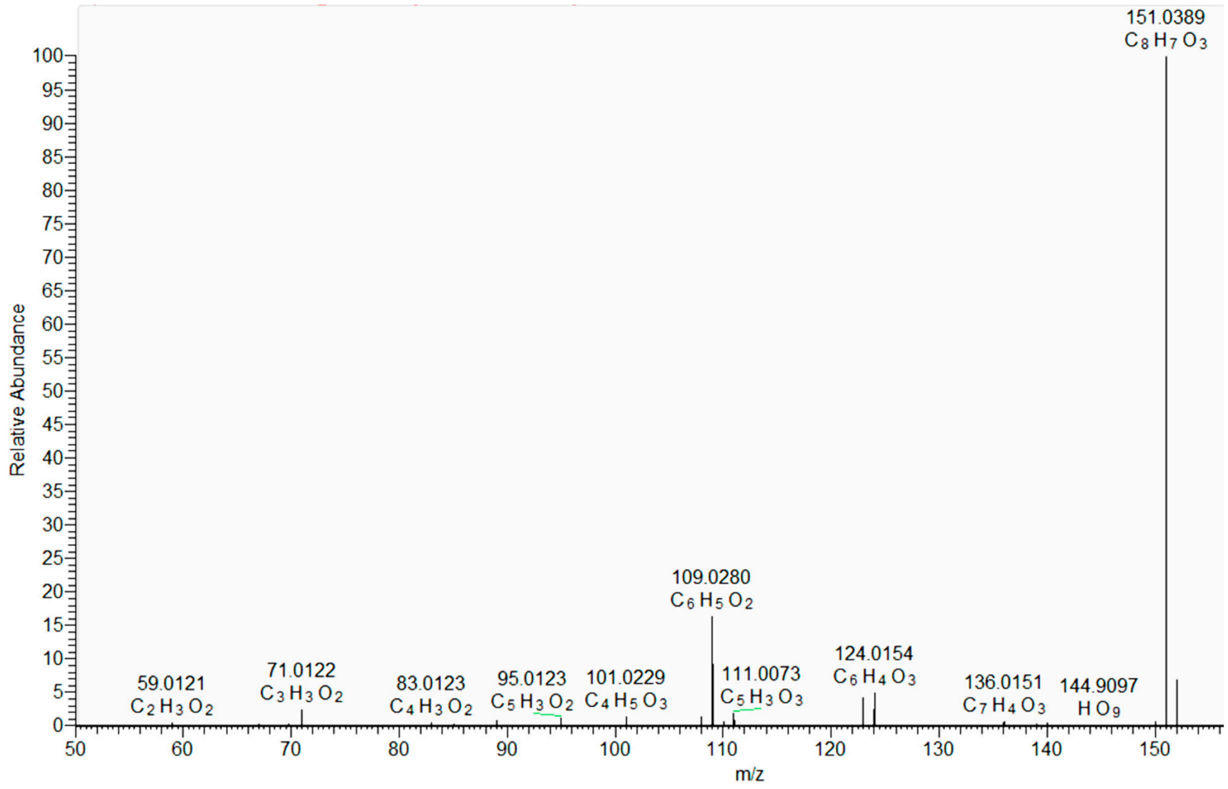

Figure S5. ESI-MS/MS spectrum of *p*-hydroxyphenyl acetic acid (21) at *m/z* 151.0400 (mass accuracy 5 ppm) (for numbers and fragmentation patterns, see Table 1).

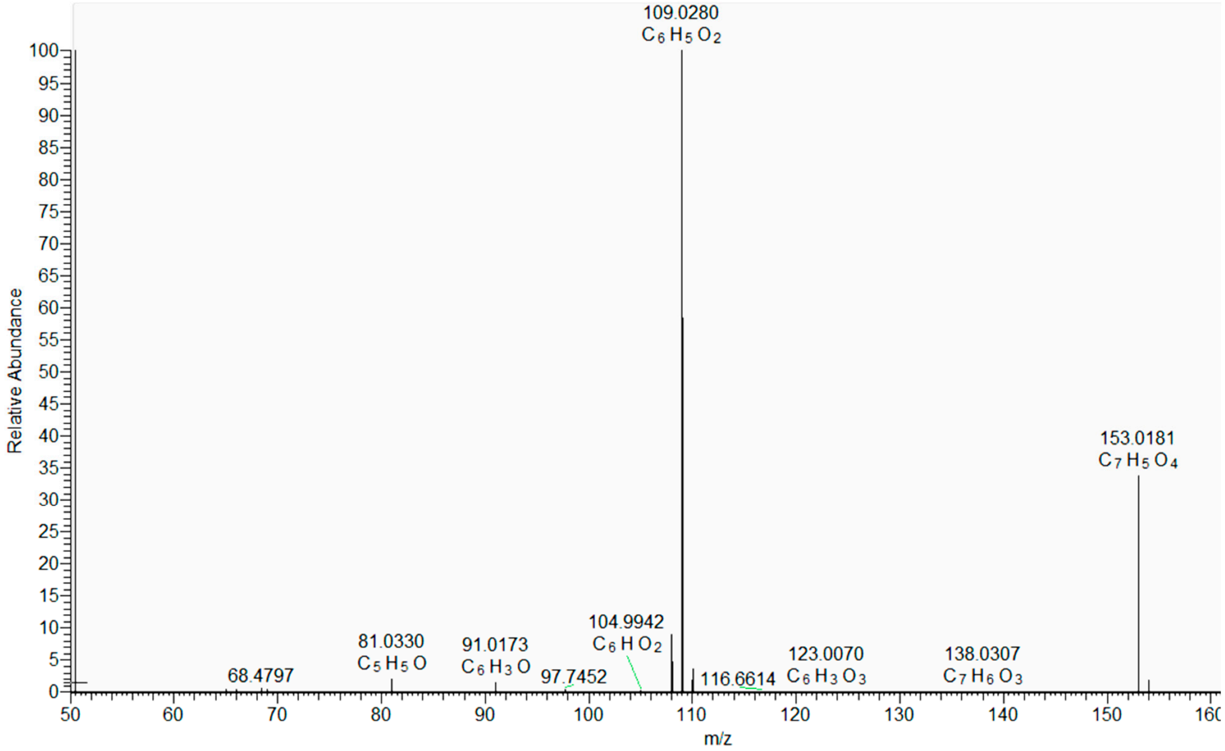

Figure S6. ESI-MS/MS spectrum of gentisic acid (22) at *m/z* 151.0400 (mass accuracy 5 ppm) (for numbers and fragmentation patterns, see Table 1).

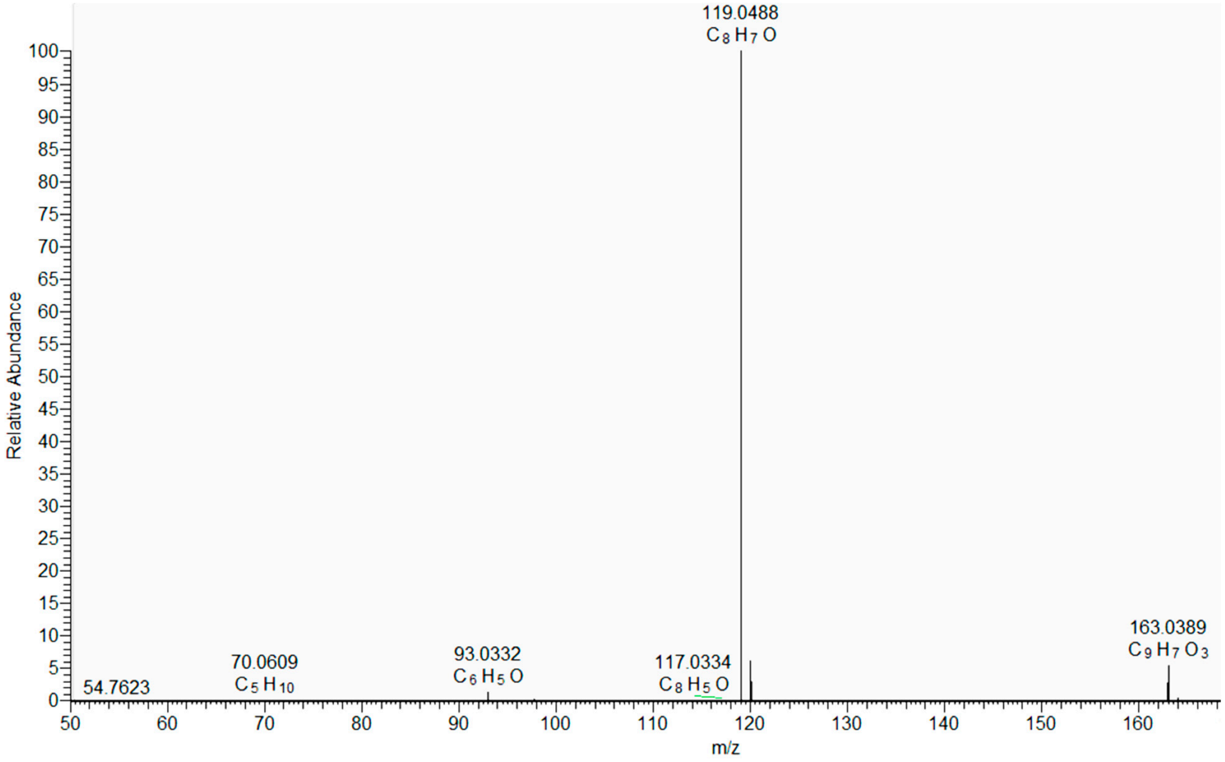

Figure S7. ESI-MS/MS spectrum of *o*-coumaric acid (23) at *m/z* 163.0401 (mass accuracy 5 ppm) (for numbers and fragmentation patterns, see Table 1).

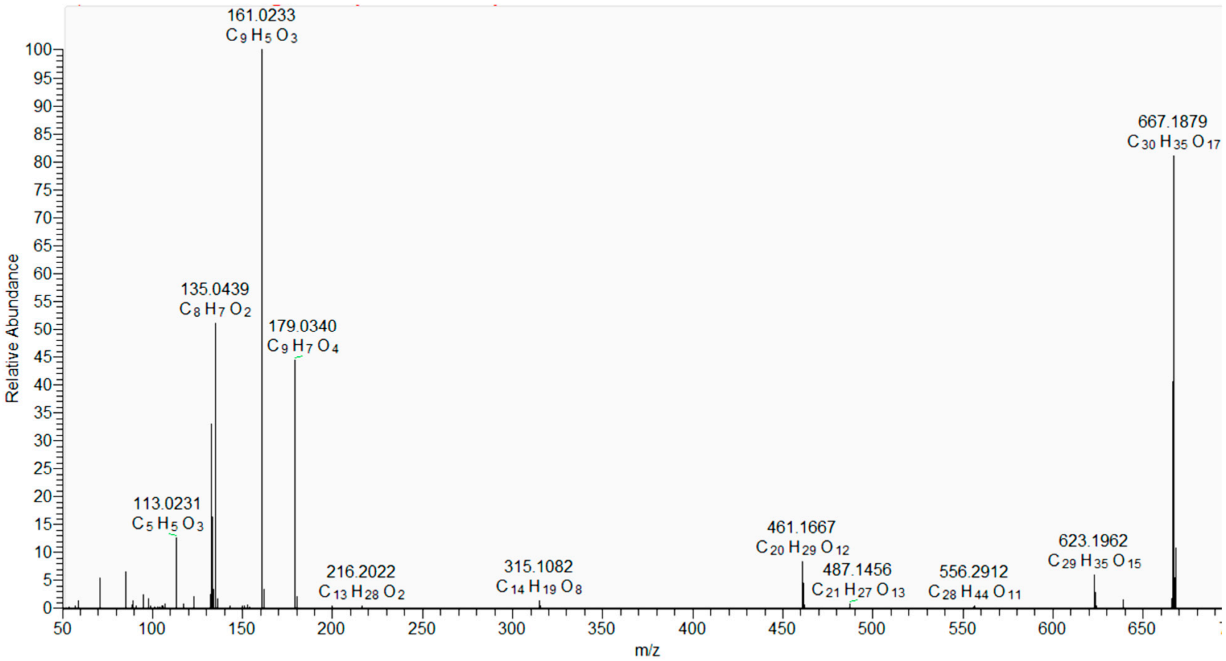

Figure S8. ESI-MS/MS spectrum of carboxyverbascoside (32) at *m/z* 667.1880 (mass accuracy 5 ppm) (for numbers and fragmentation patterns, see Table 1).

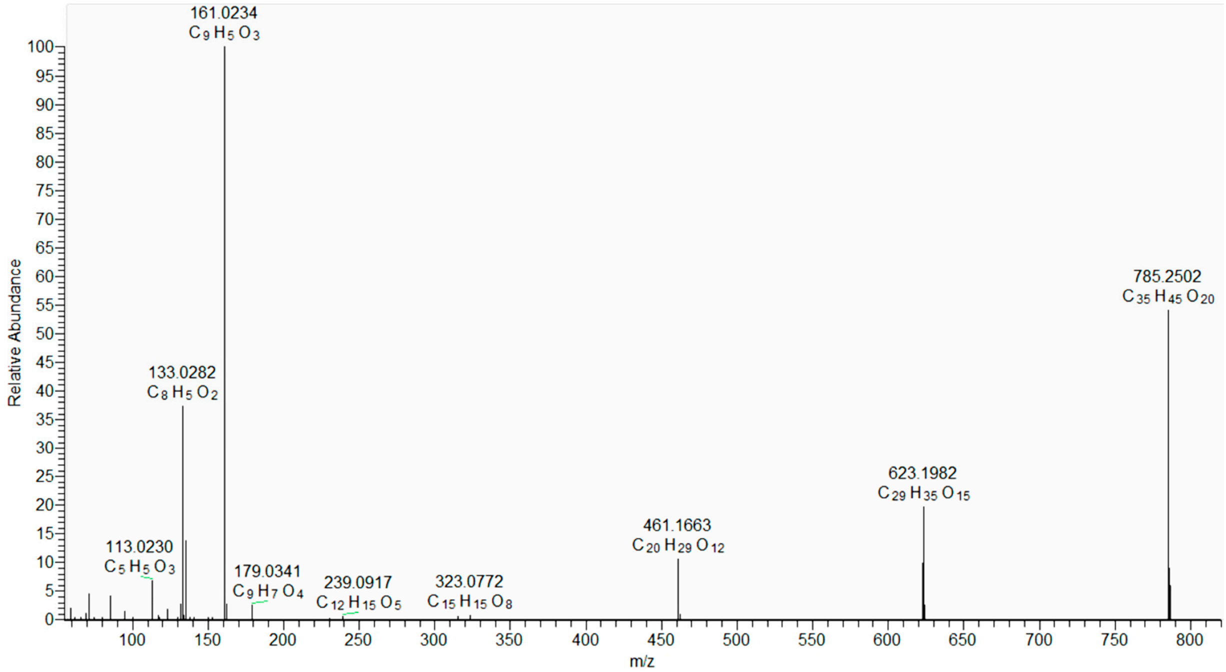

Figure S9. ESI-MS/MS spectrum of echinacoside (31) at *m/z* 785.2510 (mass accuracy 5 ppm) (for numbers and fragmentation patterns, see Table 1).

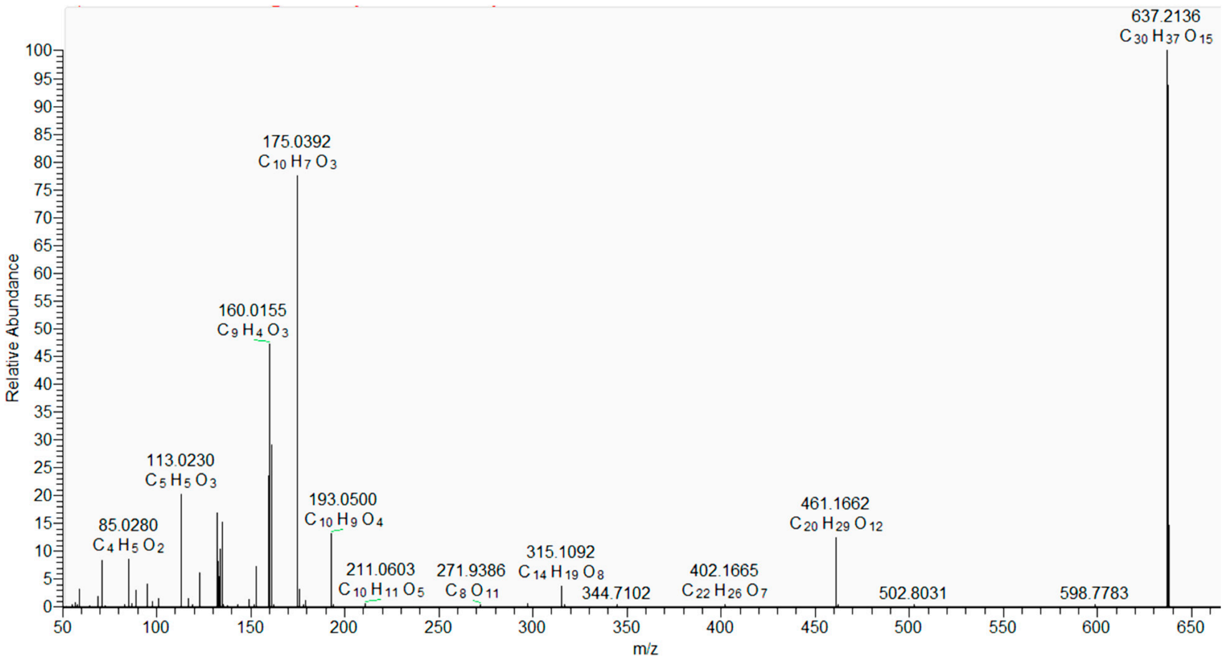

Figure S10. ESI-MS/MS spectrum of leucoseptoside A (41) at *m/z* 637.2138 (mass accuracy 5 ppm) (for numbers and fragmentation patterns, see Table 1).

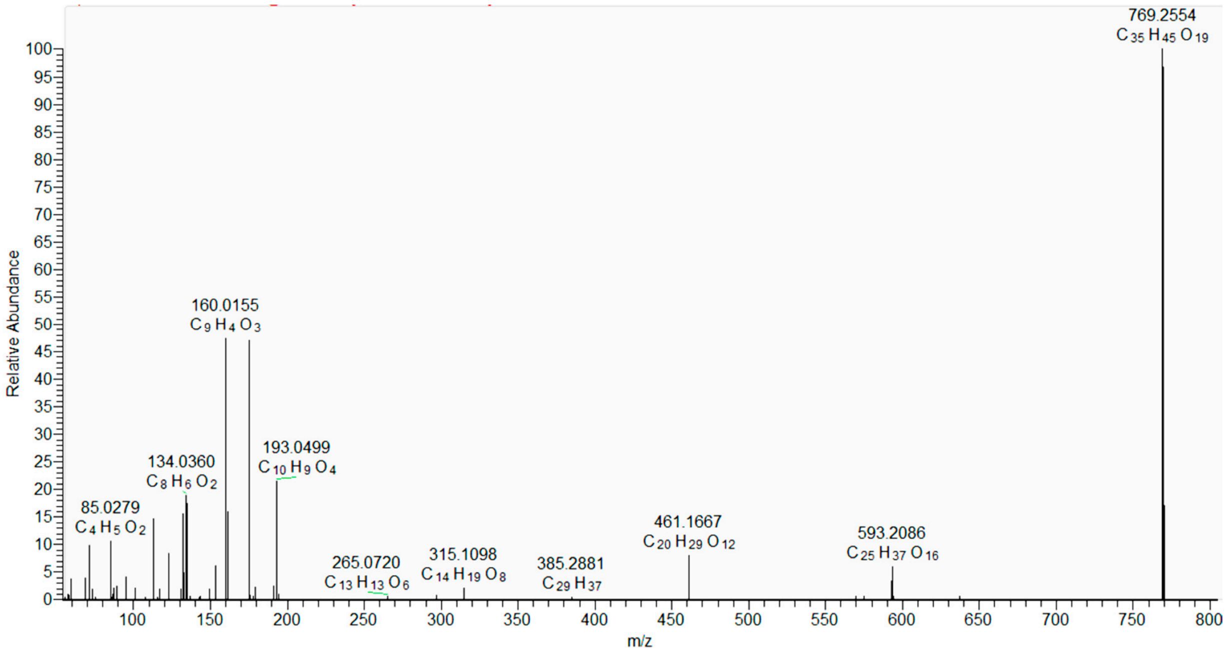

Figure S11. ESI-MS/MS spectrum of alyssonoside (40) at *m/z* 769.2561 (mass accuracy 5 ppm) (for numbers and fragmentation patterns, see Table 1).

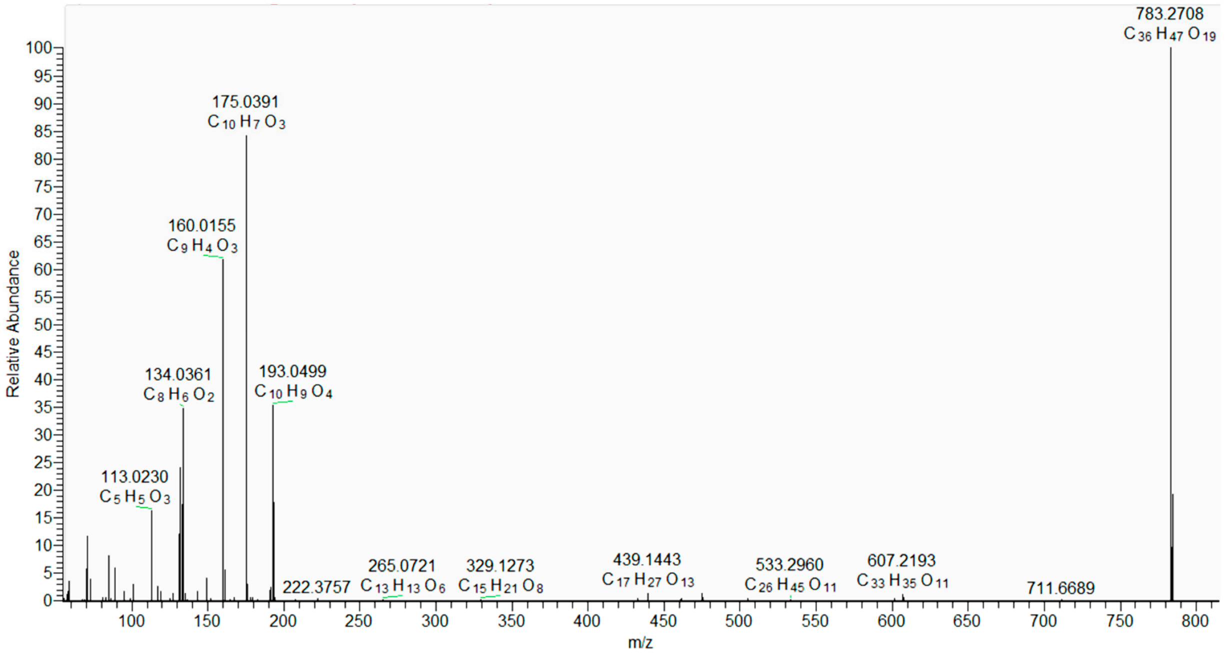

Figure S12. ESI-MS/MS spectrum of leontoside B (40) at *m/z* 783.2717 (mass accuracy 5 ppm) (for numbers and fragmentation patterns, see Table 1).

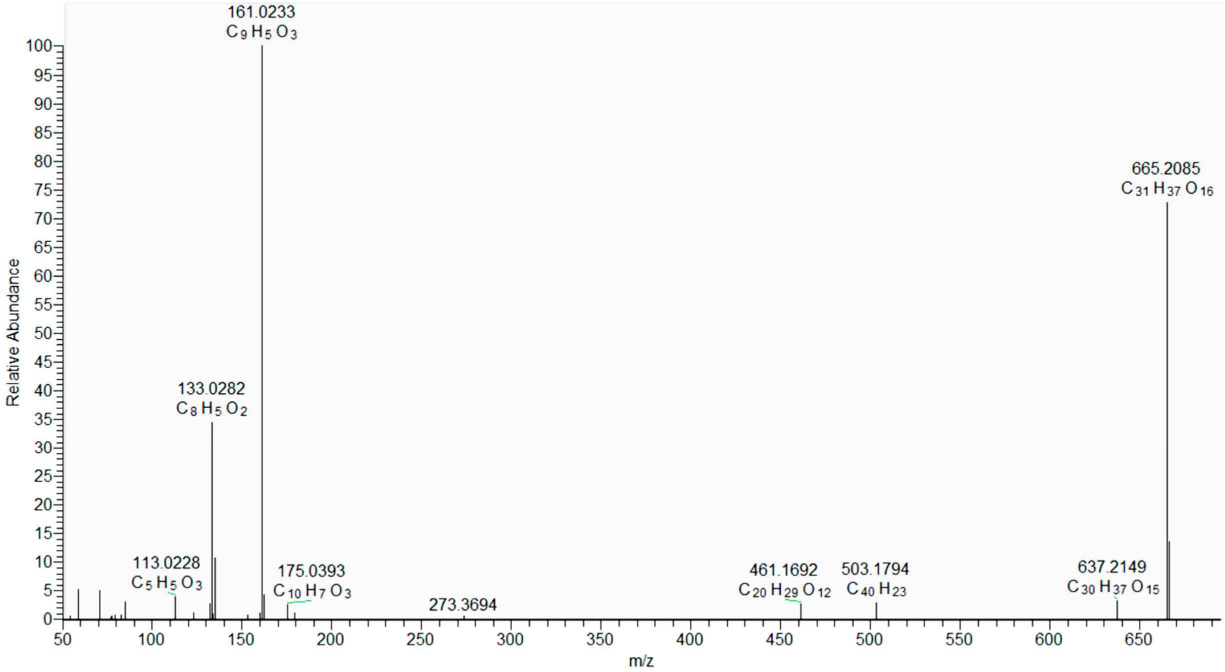

Figure S13. ESI-MS/MS spectrum of acetylvarbascoside (44) at *m/z* 665.2087 (mass accuracy 5 ppm) (for numbers and fragmentation patterns, see Table 1).

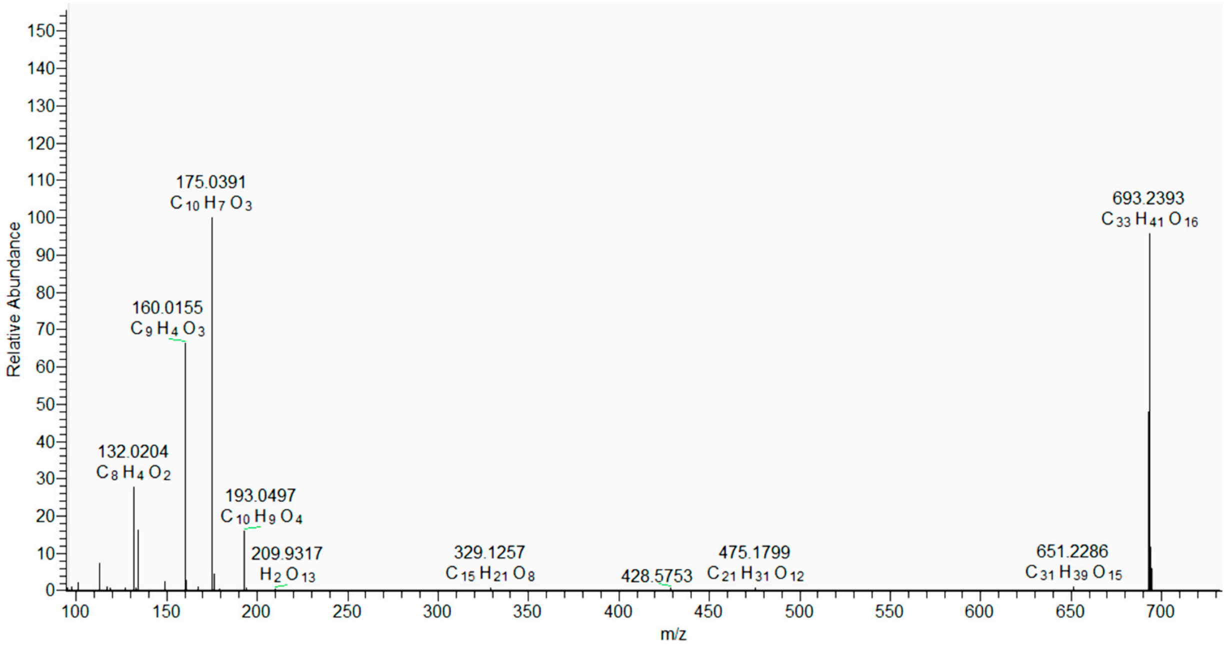

Figure S14. ESI-MS/MS spectrum of acetylmartynoside (46) at *m/z* 693.2400 (mass accuracy 5 ppm) (for numbers and fragmentation patterns, see Table 1).

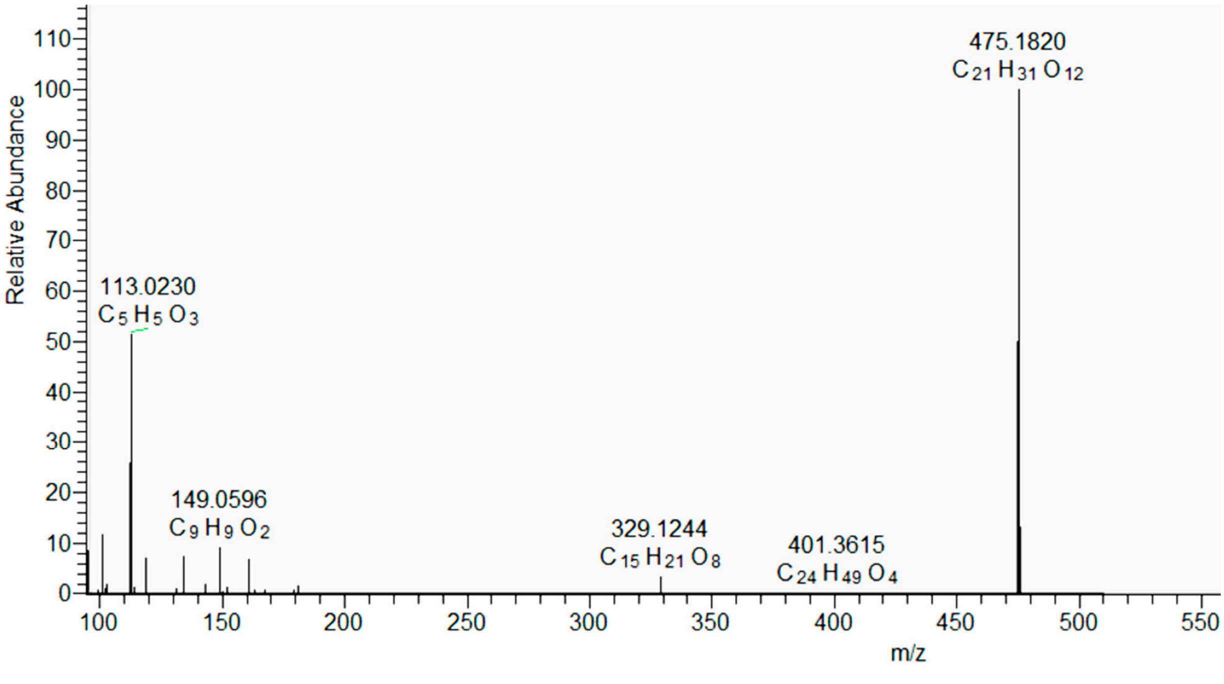

Figure S15. ESI-MS/MS spectrum of darendoside B (29) at *m/z* 475.1821 (mass accuracy 5 ppm) (for numbers and fragmentation patterns, see Table 1).

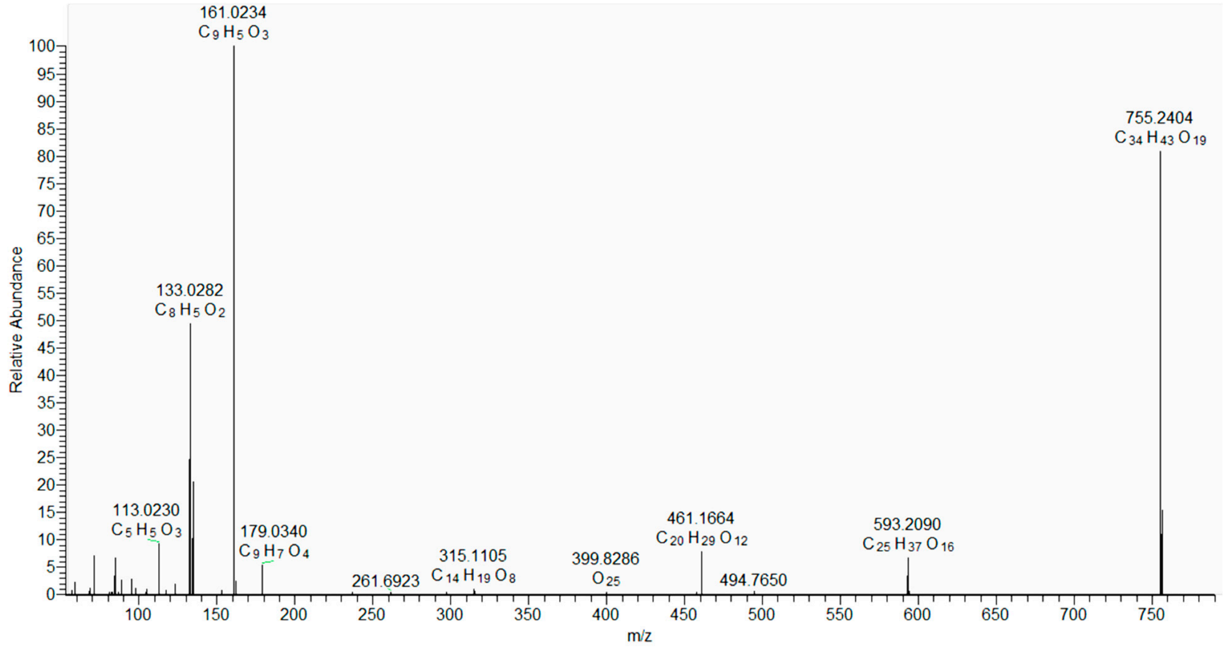

Figure S16. ESI-MS/MS spectrum of forsythoside B/samioside/lavandulifolioside (34) at *m/z* 755.2404 (mass accuracy 5 ppm) (for numbers and fragmentation patterns, see Table 1).

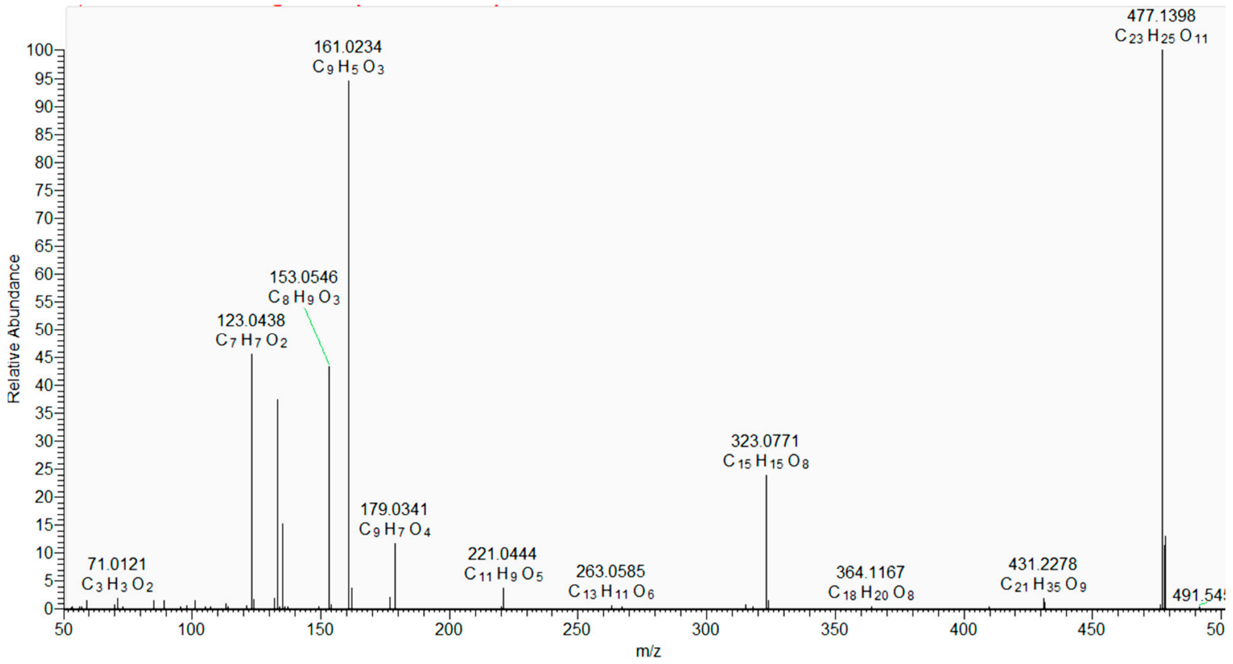

Figure S17. ESI-MS/MS spectrum of calceolarioside (37) at *m/z* 477.1402 (mass accuracy 5 ppm) (for numbers and fragmentation patterns, see Table 1).

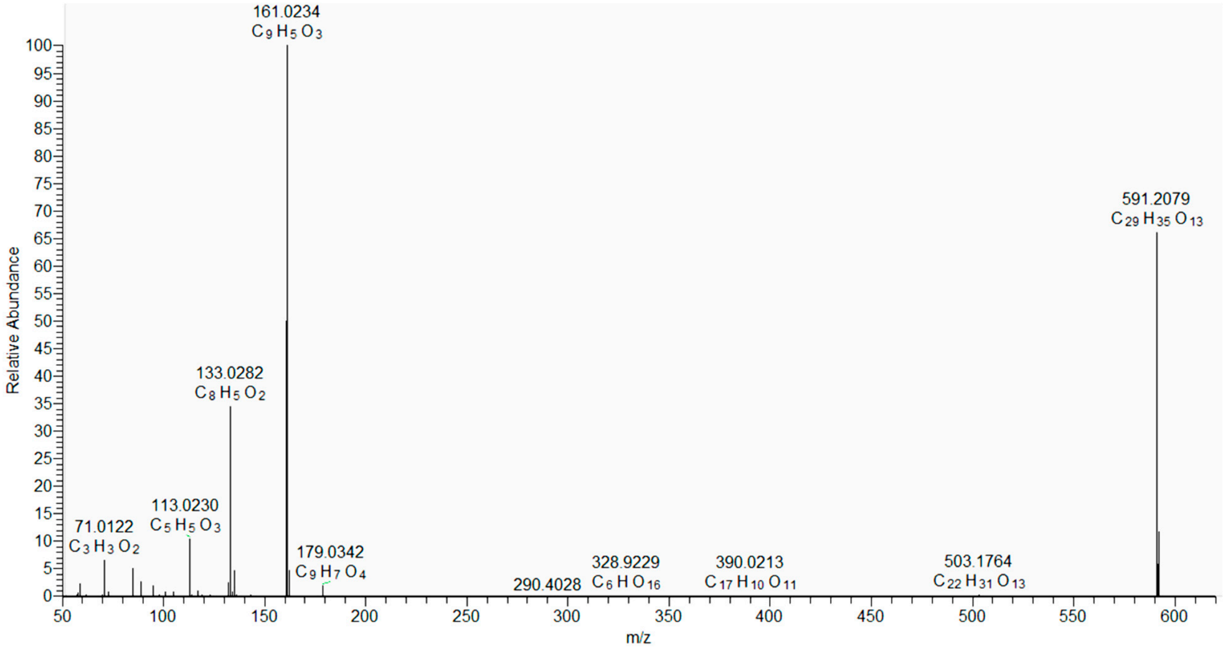

Figure S18. ESI-MS/MS spectrum of jionoside C (47) at *m/z* 591.2083 (mass accuracy 5 ppm) (for numbers and fragmentation patterns, see Table 1).

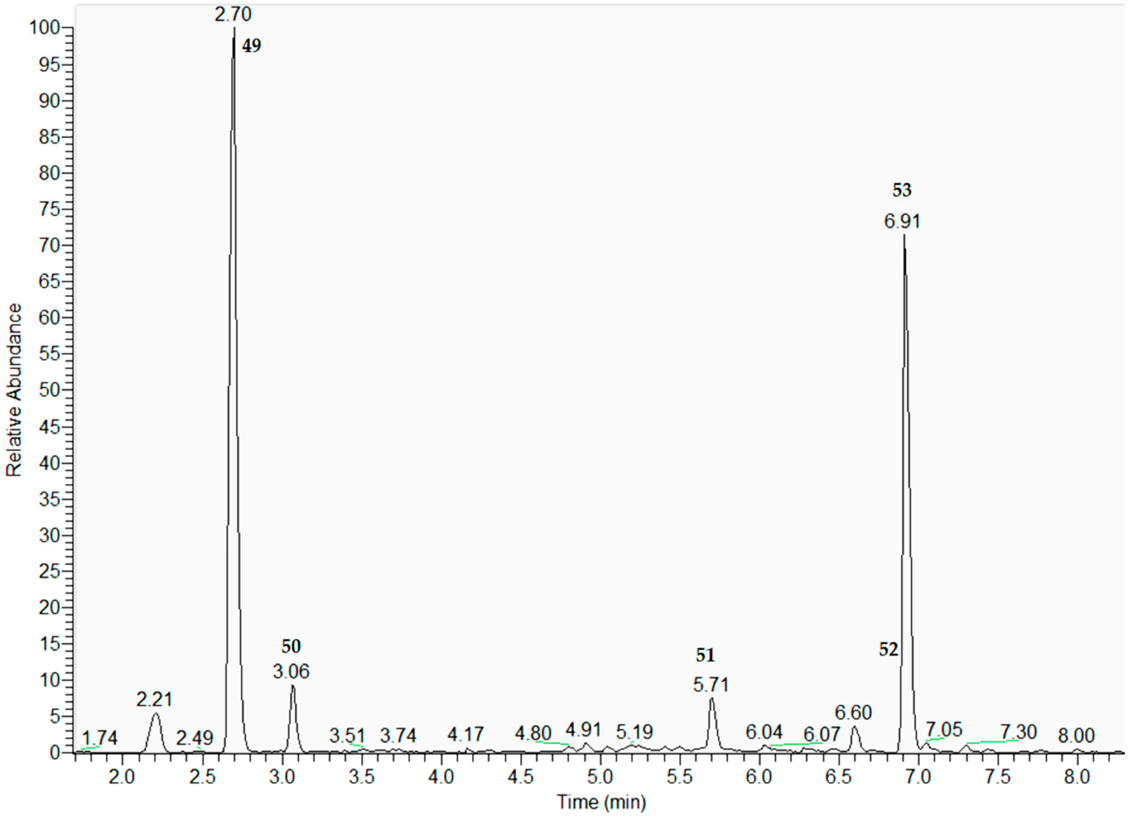

Figure S19. Extracted ion chromatogram (EIC) of iridoid and lignan glycosides. EIC was proceed with mass tolerance of 5 ppm (for numbers and fragmentation patterns, see Table 1).

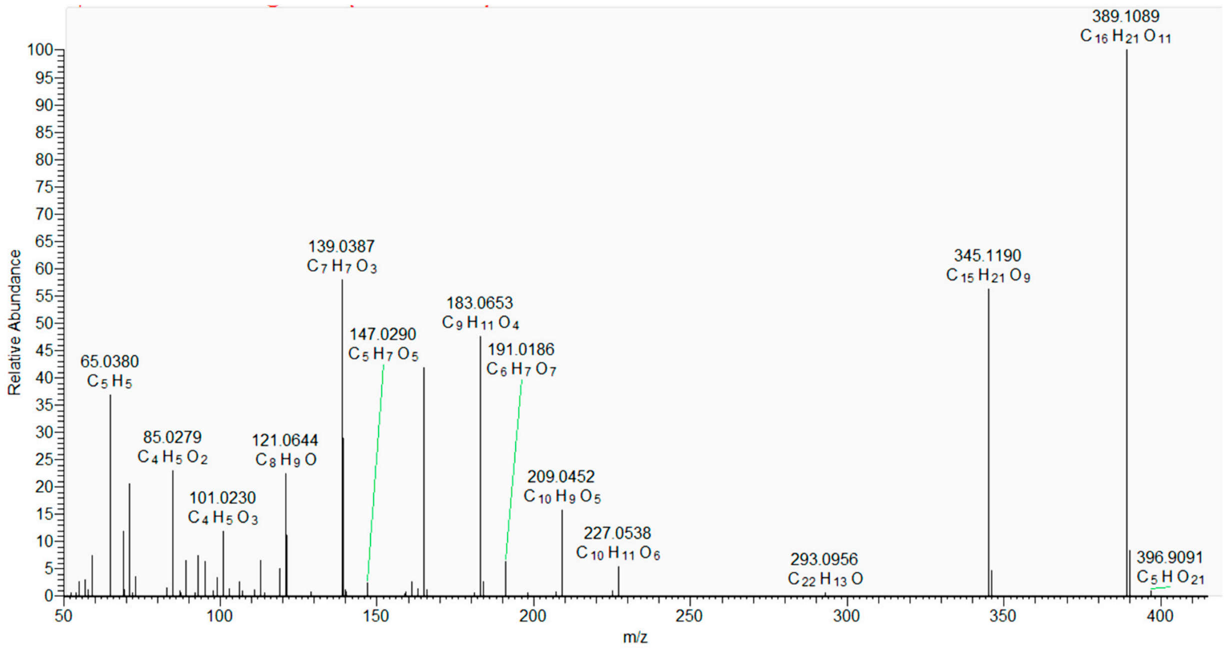

Figure S20. ESI-MS/MS spectrum of monotropein (50) at *m*/*z* 389.1089 (mass accuracy 5 ppm) (for numbers and fragmentation patterns, see Table 1).

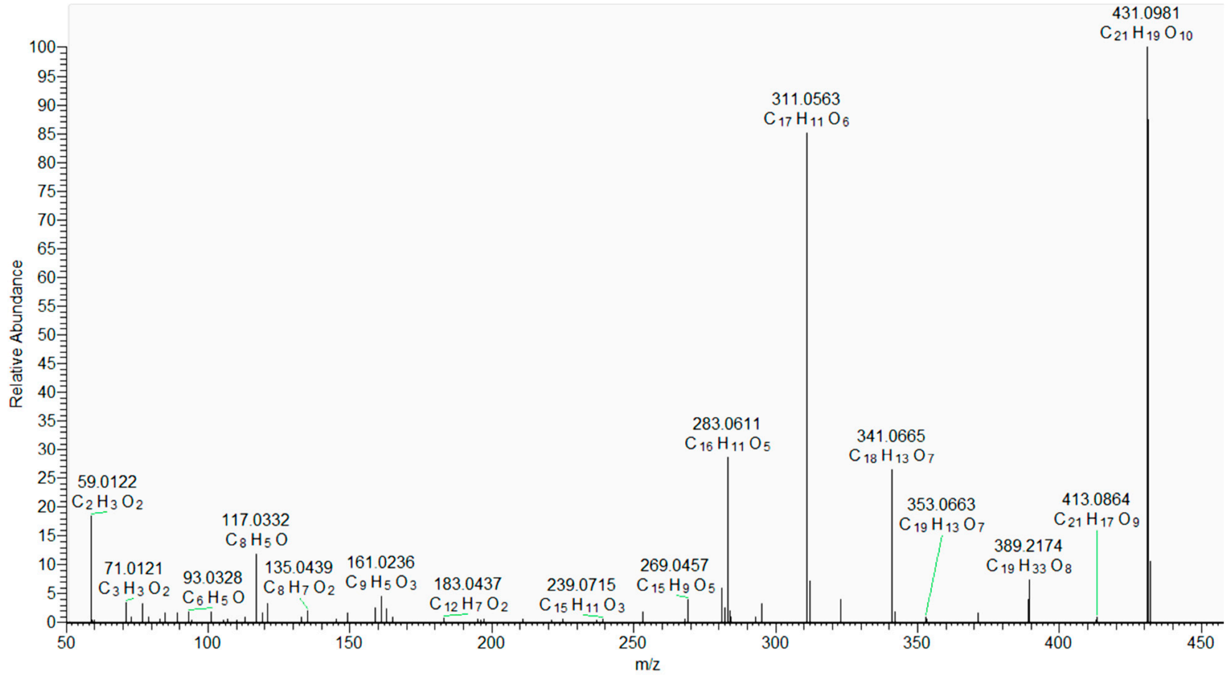

Figure S21. ESI-MS/MS spectrum of isovitexin (57) at *m*/*z* 431.0984 (mass accuracy 5 ppm) (for numbers and fragmentation patterns, see Table 1).

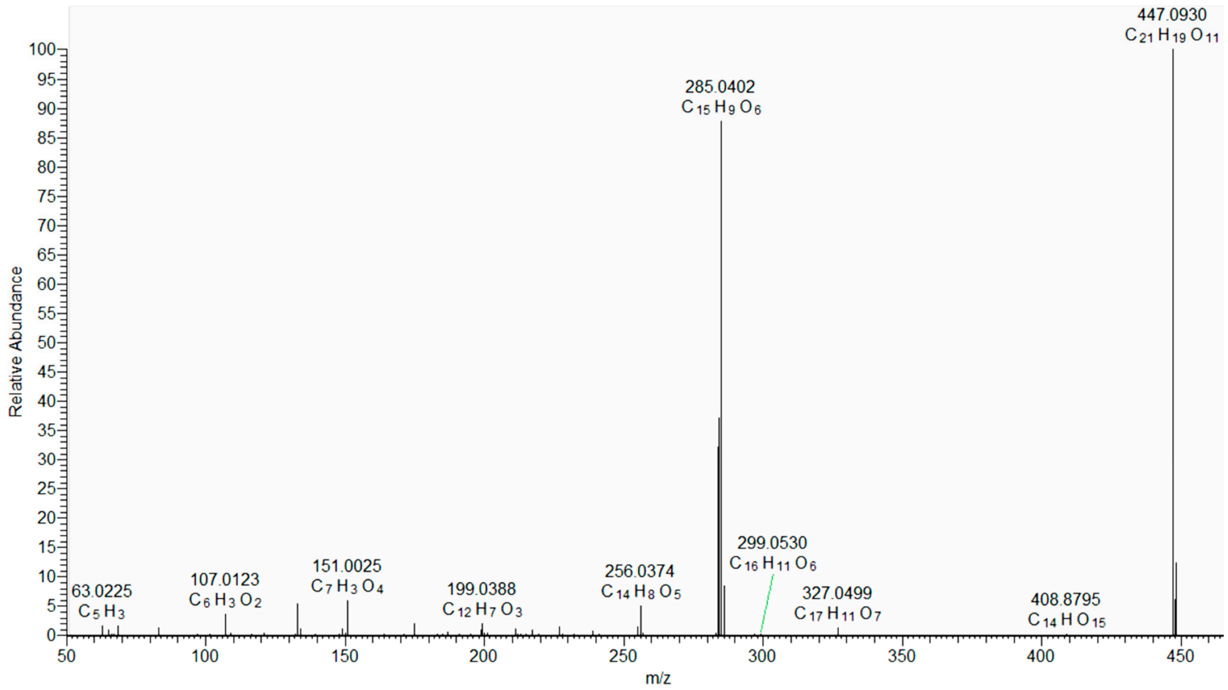

Figure S22. ESI-MS/MS spectrum of luteolin 7-*O*-glucoside (61) at *m*/*z* 447.0933 (mass accuracy 5 ppm) (for numbers and fragmentation patterns, see Table 1).

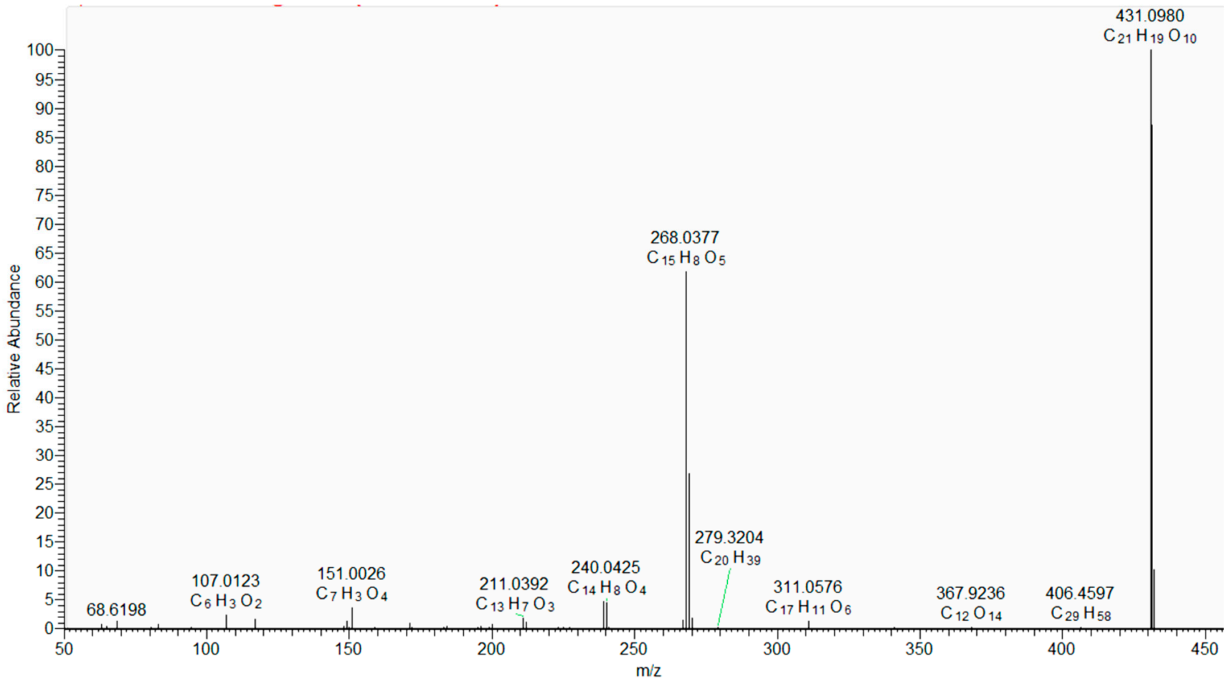

Figure S23. ESI-MS/MS spectrum of apigenin 7-*O*-glucoside (63) at *m*/*z* 431.0984 (mass accuracy 5 ppm) (for numbers and fragmentation patterns, see Table 1).

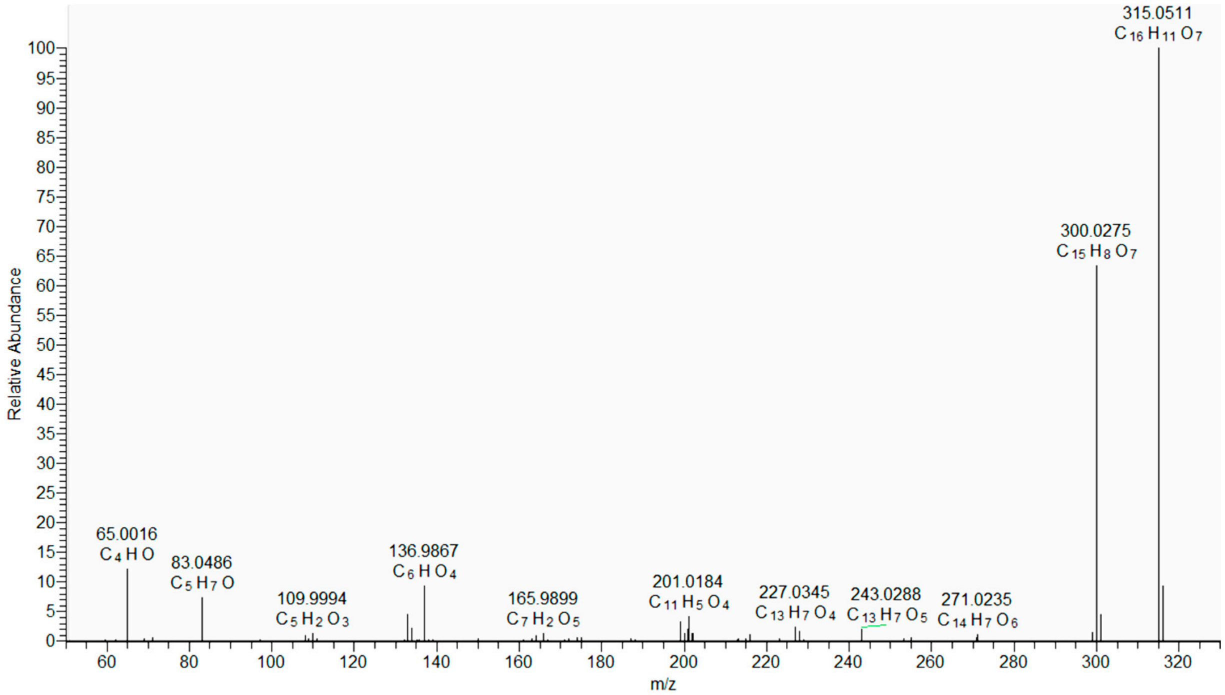

Figure S24. ESI-MS/MS spectrum of nepetin (64) at *m/z* 315.0510 (mass accuracy 5 ppm) (for numbers and fragmentation patterns, see Table 1).

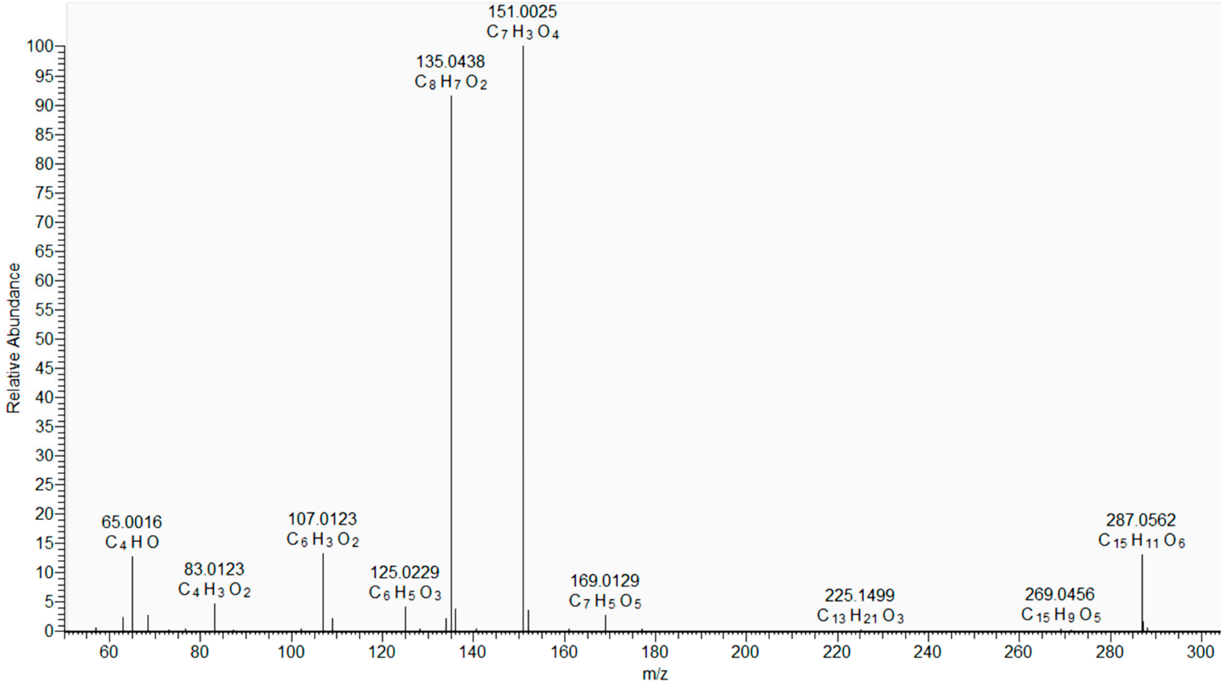

Figure S25. ESI-MS/MS spectrum of eriodictyol (66) at *m/z* 287.0561 (mass accuracy 5 ppm) (for numbers and fragmentation patterns, see Table 1).

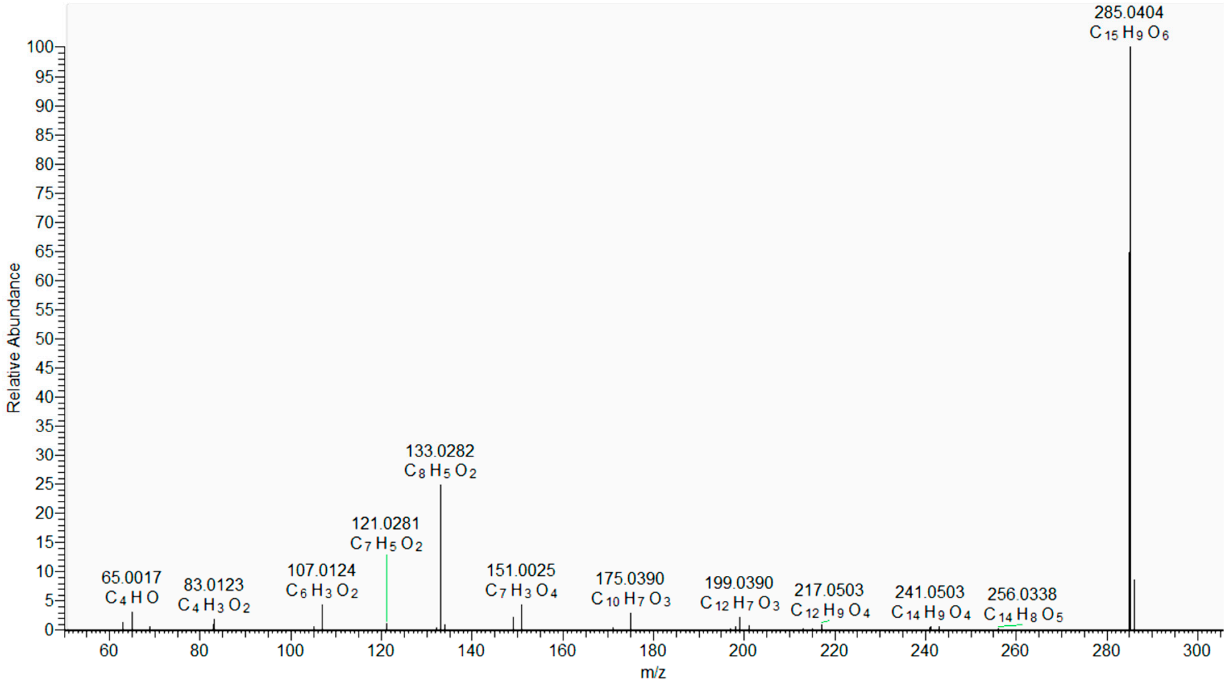

Figure S26. ESI-MS/MS spectrum of luteolin (67) at *m/z* 285.0405 (mass accuracy 5 ppm) (for numbers and fragmentation patterns, see Table 1).

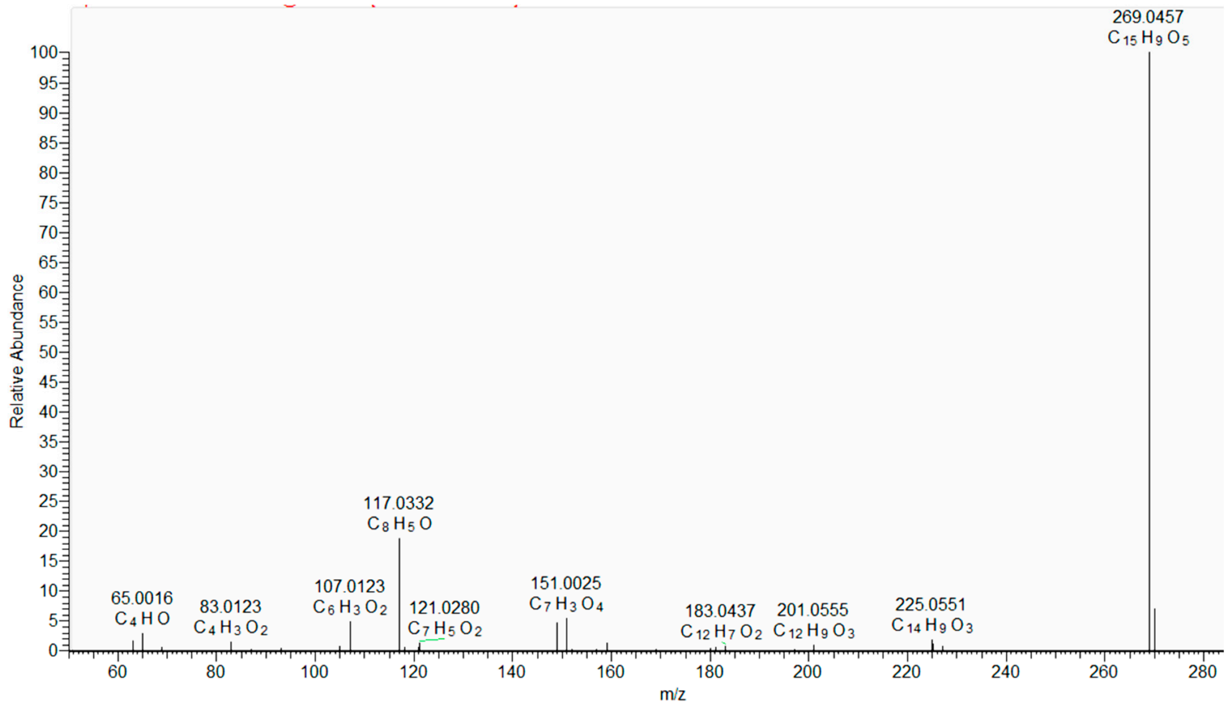

Figure S27. ESI-MS/MS spectrum of apigenin (72) at *m/z* 269.0455 (mass accuracy 5 ppm) (for numbers and fragmentation patterns, see Table 1).

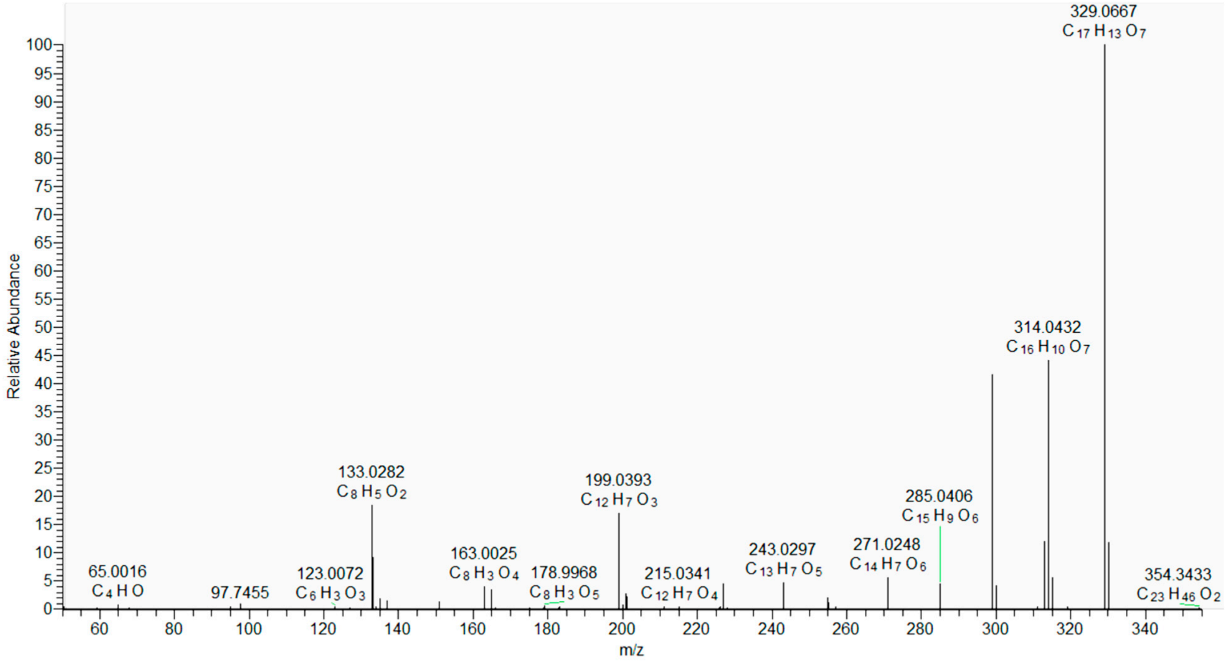

Figure S28. ESI-MS/MS spectrum of cirsiliol (74) at *m*/*z* 329.0667 (mass accuracy 5 ppm) (for numbers and fragmentation patterns, see Table 1).

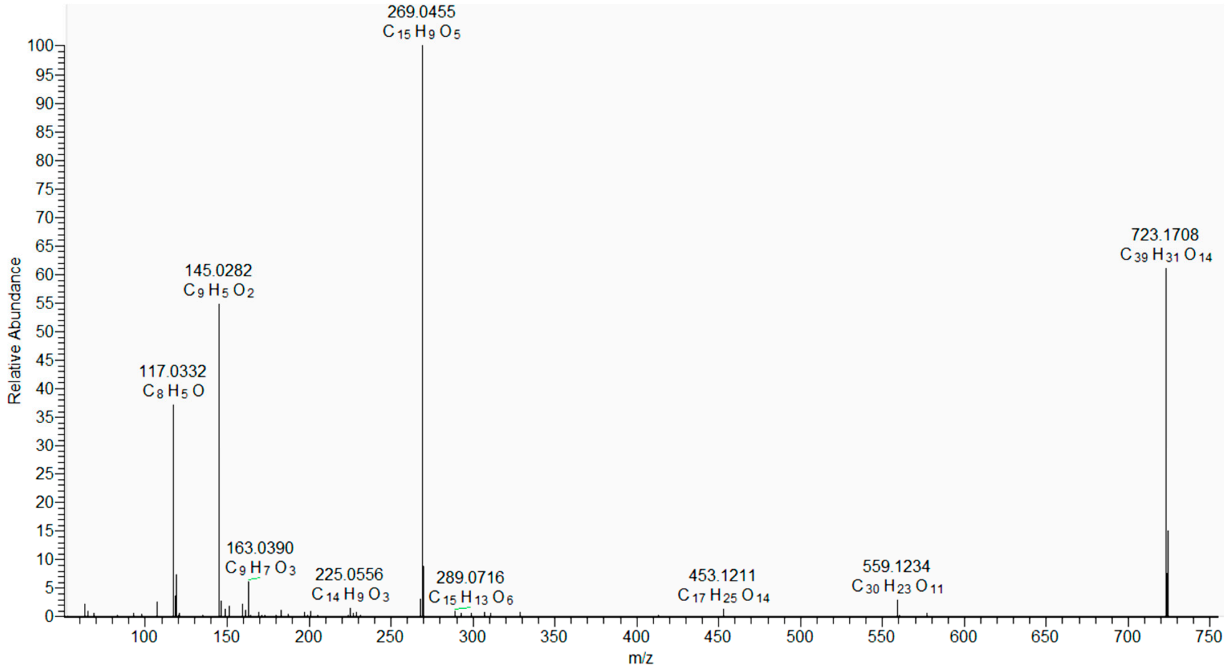

Figure S29. ESI-MS/MS spectrum of apigenin 7-*O*-dicoumaroyl-*O*-hexoside (77) at *m*/*z* 723.1719 (mass accuracy 5 ppm) (for numbers and fragmentation patterns, see Table 1).

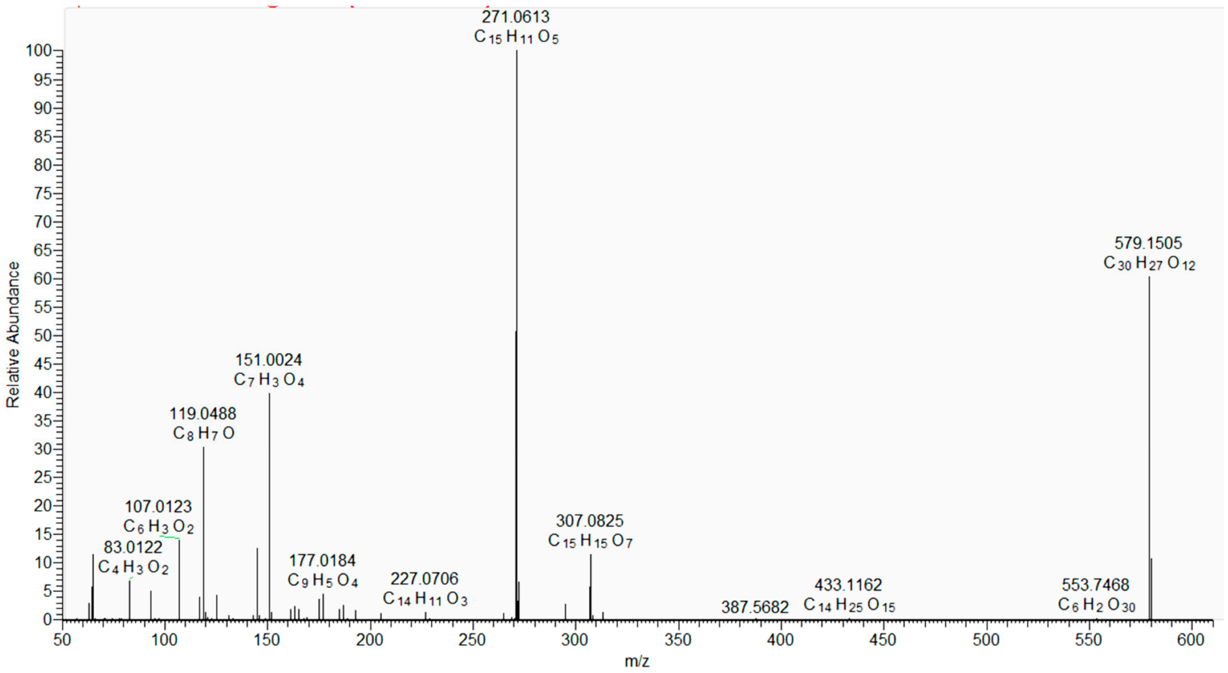

Figure S30. ESI-MS/MS spectrum of naringenin 7-*O*-coumaroyl-*O*-hexoside (71) at *m/z* 579.1508 (mass accuracy 5 ppm) (for numbers and fragmentation patterns, see Table 1).

References

1. Uysal, S.; Zengin, G.; Locatelli, M.; Bahadori, M. B.; Mocan, A.; Bellagamba, G.; De Luca, E.;Mollica, A.; Aktumsek, A., Cytotoxic and enzyme inhibitory potential of two Potentilla species(P. speciosa L. and P. reptans Willd.) and their chemical composition. *Frontiers in pharmacology* **2017,** 8, 290.

2. Grochowski, D. M.; Uysal, S.; Aktumsek, A.; Granica, S.; Zengin, G.; Ceylan, R.; Locatelli, M.;Tomczyk, M., In vitro enzyme inhibitory properties, antioxidant activities, and phytochemicalprofile of Potentilla thuringiaca. *Phytochemistry Letters* **2017,** 20, 365-372.
